# The Role of Wnt Signaling in Age‐Related Alveolar Bone Loss and Regeneration

**DOI:** 10.1111/jre.70085

**Published:** 2026-02-04

**Authors:** Hsiao H. Sung, Navya Chalamalasetty, Ali Alzainal, Hanlong Liu, Lexuan Wang, Pietra Colacrai Arikita, Ivy C. X. Wei, Hom‐Lay Wang, M. H. J. van den Bosch, Michelle S. Caird, Peter M. van der Kraan, Kenneth M. Kozloff, Esmeralda Blaney Davidson

**Affiliations:** ^1^ Orthopaedic Research Laboratories, Department of Orthopaedic Surgery University of Michigan Ann Arbor Michigan USA; ^2^ Department of Oral and Maxillofacial Surgery University of Michigan Ann Arbor Michigan USA; ^3^ Experimental Rheumatology, Department of Rheumatology Radboud University Medical Centre Nijmegen the Netherlands; ^4^ Department of Biologic and Materials Sciences & Prosthodontics University of Michigan Ann Arbor Michigan USA; ^5^ Department of Periodontics and Oral Medicine University of Michigan Ann Arbor Michigan USA

**Keywords:** aging, alveolar bone, periodontium, Wnt signaling

## Abstract

The periodontium is a uniquely dynamic tissue system requiring precise signaling for lifelong adaptation. The canonical Wnt/β‐catenin pathway is a master regulator of bone homeostasis; however, its role in the specialized environment of the alveolar bone—marked by rapid turnover, complex mechanical forces, and exposure to the oral microbiome—remains incompletely understood, particularly in the context of aging. This review critically synthesizes evidence on Wnt signaling in alveolar bone remodeling, with a focus on age‐related dysregulation, contrasting established paradigms from long bone biology with emerging oral‐tissue‐specific data. Wnt/β‐catenin signaling is essential for periodontal homeostasis, orchestrating osteoblastogenesis and mechanotransduction. Its activity is compartment‐specific within the periodontium and is potently suppressed in pathology. Key mechanisms of age‐related decline include the upregulation of Wnt antagonists (e.g., sclerostin, DKK1), cellular senescence, altered FoxO–Wnt crosstalk under oxidative stress, and impaired mechanosensing. These changes converge to disrupt regenerative capacity, tipping the balance toward net alveolar bone loss. Therapeutically, sclerostin inhibition demonstrates robust preclinical efficacy in rescuing bone loss in models of periodontitis and estrogen deficiency. However, the potential cardiovascular risks of systemic Wnt activation suggest that redirecting efforts toward localized delivery strategies could be a promising alternative. Aging induces a multifaceted suppression of regenerative Wnt signaling in the periodontium. Modulating the Wnt pathway shows great potential for oral bone regeneration. However, significant challenges exist, especially in designing local delivery systems that are both safe and effective. Overcoming these hurdles is crucial for successful clinical applications. Future research must bridge the gap between skeletal biology and direct oral‐specific investigations to enable targeted therapies that preserve periodontal health in an aging population.

## Summary

- The integrity of the periodontium, a uniquely dynamic and mechanically stressed tissue system, is fundamentally governed by Wnt/β‐catenin signaling, which orchestrates alveolar bone homeostasis and adaptation.
- Aging induces a multifaceted decline in this regenerative pathway, driven by the upregulation of antagonists like sclerostin, the accumulation of senescent cells, and the blunting of essential mechanosensory functions.
- This age‐related suppression of Wnt signaling converges with local inflammatory pathology, such as periodontitis, to shift the periodontal balance toward irreversible bone loss.
- While targeted inhibition of Wnt antagonists demonstrates robust preclinical potential for regenerating alveolar bone, the translation of this promise into safe clinical reality is challenged by the cardiovascular risks of systemic therapy, underscoring an urgent need for localized delivery strategies and dedicated research to bridge foundational skeletal biology with the unique demands of the oral environment.

## Graphical abstract

The graphical abstract summarizes key signaling pathways involved in bone formation and resorption associated with Wnt signaling across young and aged long bone and jaw tissues. Levels of evidence are indicated as robust, moderate, or emerging, reflecting the current experimental support in each context.

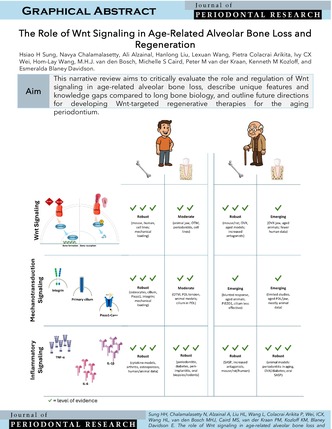

## 1 Introduction

The periodontium constitutes a dynamic, integrated tissue complex essential for oral function, comprising alveolar bone, periodontal ligament (PDL), cementum, and gingiva. Through lifelong, coordinated remodeling, this system maintains tooth anchorage and adapts to mechanical forces, environmental exposures, and injury [1, 2, 3].

The Wnt signaling pathway is a master regulator of bone formation and resorption in both systemic and oral environments [4, 5]. However, our foundational understanding of Wnt biology, and of bone adaptation more broadly, has been driven predominantly by research in long bones, where it orchestrates osteoblast activity, mechanotransduction, and fracture repair [6, 7, 8]. The periodontium presents a fundamentally distinct ecological niche: characterized by rapid turnover rates [9, 10], complex multidirectional mechanical stimuli [11, 12], and tissues that are continuously exposed to the oral microbiome and inflammatory challenges [13, 14]. Consequently, the direct extrapolation of molecular mechanisms and therapeutic strategies from the appendicular skeleton to the oral cavity is fraught with challenge and requires rigorous, tissue‐specific validation.

Aging critically disrupts the homeostatic equilibrium of this specialized system. Systemic hallmarks of aging, including declines in stem cell function, Wnt signaling efficacy, and bone quality, combine with local risk factors, such as malocclusion and periodontitis, to disrupt normal regenerative responses and tip the balance toward net bone loss [6, 7, 15, 16]. Clinically, these changes manifest as increased tooth mobility, diminished masticatory function, and heightened vulnerability to oral disease in older adults [14]. The periodontium thus represents a critical, and vulnerable, interface where systemic aging and local pathology intersect.

Emerging research in periodontal biology now highlights the need for targeted investigation into canonical and non‐canonical Wnt pathways, local antagonists like sclerostin and Dickkopf‐related protein 1 (DKK1), and regenerative strategies tailored to the periodontium itself [17, 18, 19, 20, 21]. While therapeutic advances inspired by osteoporosis management offer promising approaches for dental and periodontal conditions, significant translational hurdles remain. These include fragmented direct evidence from oral tissues, safety concerns with systemic therapy, technical barriers to local delivery, and a limited number of clinical trials specific to the oral environment.

This review critically synthesizes current evidence on the role of Wnt signaling within the periodontium, with a focused emphasis on alveolar bone remodeling and its dysregulation with age. By deliberately contrasting established paradigms from long bone biology with emerging, direct data from oral tissues, we aim to: (1) clarify tissue‐specific regulatory mechanisms, (2) illuminate critical translational gaps, and (3) propose a refined roadmap for future Wnt‐targeted therapeutic innovation. Advancing this field is essential not only for preserving oral health, but also for improving systemic health and quality of life in aging populations (Tables 1 and 2).

**TABLE 1:** Age‐related alveolar bone loss: pathophysiological cascade and Wnt signaling dysregulation.

Factor	Effect of aging	Wnt pathway role and mechanism
Bone quantity and quality	Bone mineral density, height, and width decrease; trabecular structure weakens	Aging reduces canonical Wnt signaling → decreased osteoblast‐driven bone formation and compromised maintenance
Osteoblast function	Number and activity decline; differentiation and regenerative capacity diminish	Wnt/β‐catenin is a key promoter of osteoblast differentiation and activity; this pro‐formation signal is dampened with age
Osteoclast activity	Resorptive activity often increases, shifting remodeling balance toward net loss	Reduced Wnt signaling lowers OPG and increases RANKL expression → favors increased osteoclastogenesis and bone resorption
PDL and extracellular matrix (ECM)	PDL space narrows; collagen integrity declines; tissue loses elasticity	Impaired Wnt signaling and reduced DEL‐1 expression compromise PDL fibroblast function and ECM repair/regeneration
Local inflammatory milieu (“Inflammaging”)	Chronic, low‐grade inflammation increases pro‐resorptive cytokines (e.g., TNF‐α, IL‐1β, IL‐6)	Inflammatory cytokines upregulate secreted Wnt inhibitors (DKK1, sclerostin) → further suppress bone formation
Key molecular shift	↑ Expression of DKK1, sclerostin, SHN3; ↓ Expression of Klotho	DKK1 and sclerostin directly inhibit Wnt/β‐catenin signaling; SHN3 promotes sclerostin; Klotho decline removes a protective Wnt‐enhancing effect
Tissue repair and regeneration capacity	Healing after extraction, periodontal defect, or implant placement is slower and less predictable	Exogenous Wnt activation (e.g., L‐WNT3A) or neutralization of inhibitors (e.g., sclerostin antibody) can rescue bone healing in aged models
Therapeutic implication	Age‐related bone loss requires targeted intervention to restore anabolic signaling	Strategy: antagonize endogenous inhibitors (anti‐sclerostin/DKK1) or provide Wnt agonists to re‐establish a pro‐formative microenvironment

Abbreviations: DKK1: Dickkopf‐1; IL: interleukin; OPG: osteoprotegerin; PDL: periodontal ligament; RANKL: receptor activator of nuclear factor κB ligand; TNF‐α: tumor necrosis factor‐alpha.

**TABLE 2:** Sources of mechanistic evidence of Wnt signaling in the periodontium.

Mechanism/pathway	Evidence source	Direct/extrapolated
Canonical Wnt signaling in osteoblasts [1, 2, 3]	Long bone (mouse, human)	Extrapolated
Canonical Wnt/β‐catenin in alveolar bone	Periodontium (rodent, human)	Direct
Sclerostin antagonism	Long bone; periodontium (rodent)	Both
Mechanotransduction (Piezo1, integrins, cilia)	Periodontium (rodent, cell lines)	Direct/partial
Non‐canonical Wnt signaling	Long bone; periodontium (rodent)	Both
FoxO–Wnt crosstalk	Long bone; emerging in periodontium	Extrapolated/emerging
Senescence/SASP impact on Wnt	Long bone; periodontium (human, rodent)	Both

*Note:* This table summarizes the origins and types of mechanistic evidence underpinning current understanding of Wnt signaling pathways in the periodontium. Mechanisms are categorized by their biological context, indicating whether supporting data comes from long bone models, direct studies in periodontal tissues, or extrapolation.

## 2 Wnt Signaling and Periodontium

### 2.1 Periodontium: Alveolar Bone Structure and Remodeling

Alveolar bone is a core component of the periodontium, forming the tooth‐supporting sockets within the jaws [22, 23]. Its intimate functional integration with the PDL results in one of the most dynamic osseous environments in the body, characterized by rapid turnover rates [24, 25, 26]. This high plasticity is enabled by a specialized, compartmentalized architecture designed for both force transmission and metabolic support.

Closest to the tooth root lies the bundle bone, a thin, specialized layer distinguished by the insertion of Sharpey's fibers from the PDL [1, 27, 28]. This interface plays a crucial role in mechanotransduction, allowing the bone to rapidly adapt to varying occlusal forces [1, 11, 12, 27, 29].

Beneath the bundle bone are the cortical and trabecular compartments, which provide essential structural reinforcement while serving as a metabolic reservoir to support continuous remodeling [22, 23, 30].

**FIGURE 1:**
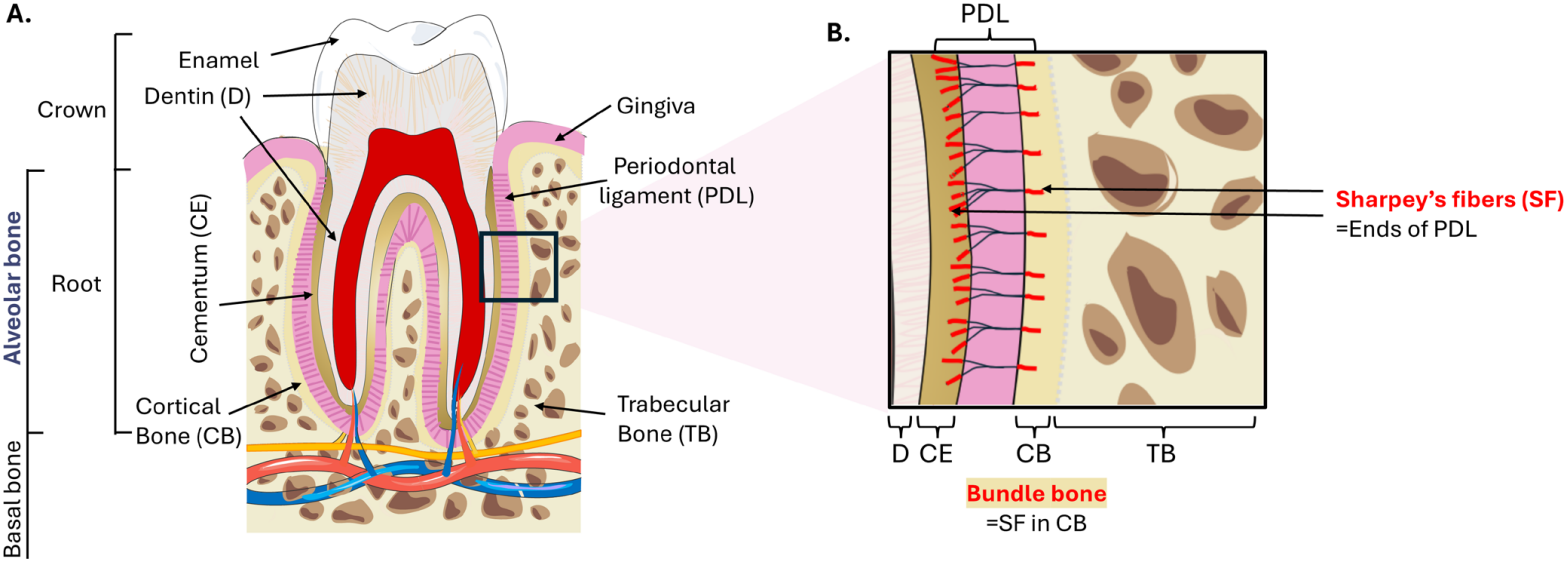
Alveolar bone structure. Alveolar bone is a specialized osseous tissue that forms the tooth sockets (alveoli) within the maxilla and mandible, providing structural support and anchorage for teeth. As part of the periodontium, it interacts dynamically with the gingiva, periodontal ligament (PDL), and cementum to maintain tooth position and masticatory function. Structurally, it comprises two key compartments: (A) the underlying cortical and trabecular bone, which provides structural reinforcement and metabolic support and (B) the bundle bone, which interfaces with the PDL via Sharpey's fibers and facilitates force transmission.

Through this integrated structure, the alveolar bone is equipped to translate mechanical signals into anabolic or catabolic responses, a defining feature that sets it apart from the long bones elsewhere in the skeleton [16, 23, 26, 31] (Figure 1).

### 2.2 Core Canonical Wnt/β‐Catenin Signaling in the Periodontium

Bone homeostasis within the periodontium is governed by the canonical Wnt/β‐catenin pathway, a mechanism conserved yet distinctly regulated in this environment. Activation begins when Wnt ligands (e.g., Wnt3a, Wnt10b) bind to Frizzled and LRP5/6 co‐receptors, triggering phosphorylation of LRP5/6. This event inhibits a cytoplasmic destruction complex, comprising Axin, Adenomatous Polyposis Coli (APC), and Glycogen Synthase Kinase‐3β (GSK‐3β), that normally targets β‐catenin for proteasomal degradation. Consequently, β‐catenin accumulates, translocates to the nucleus, and partners with TCF/LEF transcription factors to drive the expression of osteogenic genes (Runx2, COL1A1) and production of Osteoprotegerin (OPG), which inhibits RANKL‐mediated osteoclastogenesis [2, 4, 29, 32, 33].

While this core pathway is evolutionarily conserved, its regulation within the periodontium exhibits critical tissue‐specific nuances. It is essential to note that foundational knowledge of the Wnt/β‐catenin destruction complex, receptor activation, and downstream transcription is derived overwhelmingly from studies in long bones and cancer models. Direct validation of these precise molecular interactions within the unique cellular milieu of the periodontium—particularly in human tissues—remains an important, ongoing effort [16, 31].

**FIGURE 2:**
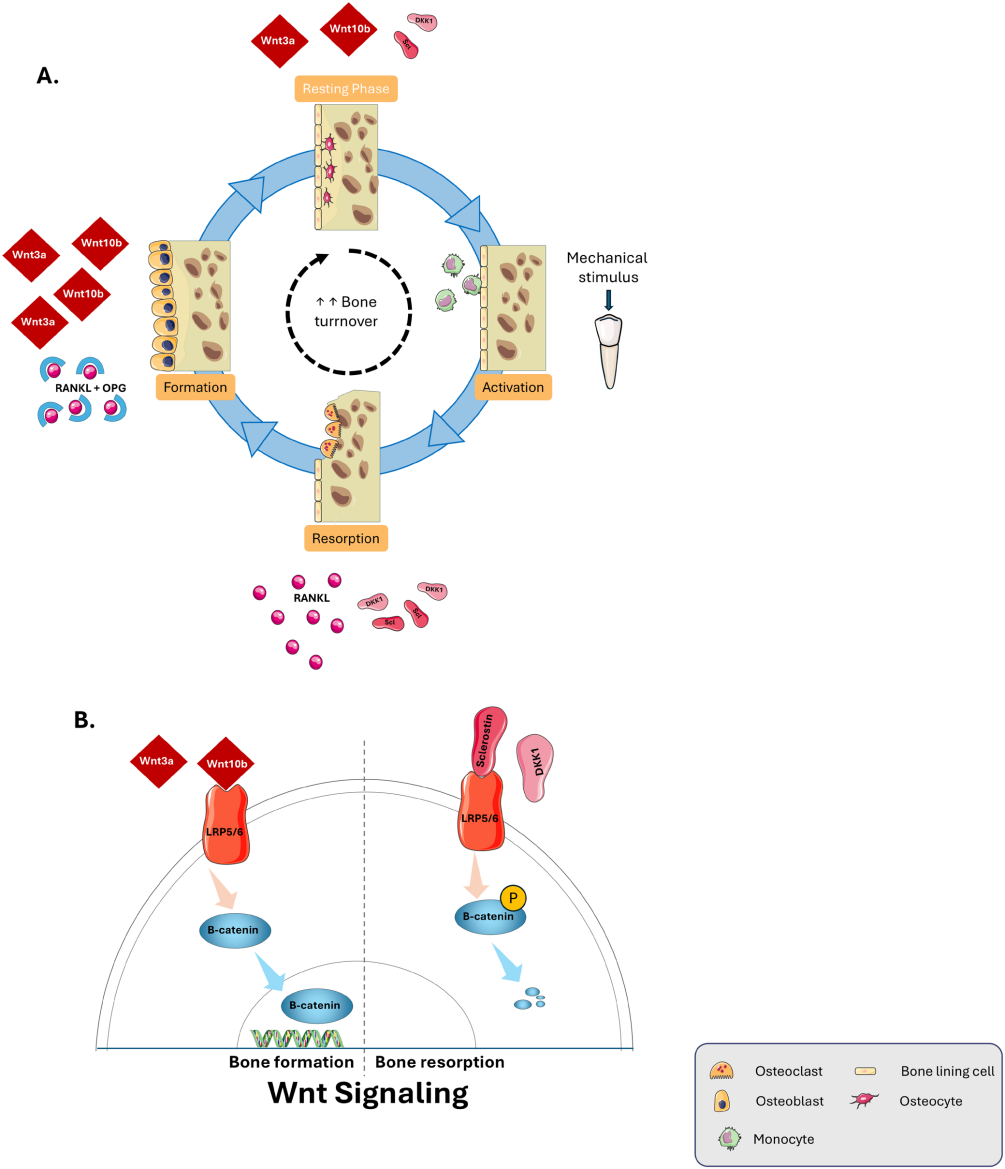
Wnt/β‐catenin signaling regulates alveolar bone remodeling in response to mechanical stimuli. (A) Alveolar bone undergoes rapid and dynamic remodeling due to its specialized microstructure and sensitivity to mechanical forces. Mechanical stimulation activates Wnt signaling, with Wnt ligands (such as Wnt3a and Wnt10b) binding to Frizzled receptors and LRP5/6 co‐receptors, thereby preventing degradation of β‐catenin. (B) Stabilized β‐catenin accumulates and translocates into the nucleus, where it promotes transcription of osteogenic genes, enhances osteoblast differentiation, and suppresses osteoclast activity. Conversely, Wnt signaling inhibitors such as sclerostin and Dkk1 bind to Frizzled and LRP5/6, leading to β‐catenin phosphorylation and degradation. Overall, the Wnt/β‐catenin pathway is central to maintaining alveolar bone homeostasis. It enables adaptive remodeling in response to mechanical (occlusal) forces, balancing bone formation and resorption and helping preserve periodontal integrity.

Direct periodontal evidence confirms this pathway's central role. Mechanical stimulation from mastication rapidly activates canonical Wnt signaling in alveolar bone [25, 34, 35]. Furthermore, genetic ablation of the Wnt antagonist sclerostin leads to excessive alveolar bone accumulation [36], while human biopsies from periodontitis patients show elevated levels of sclerostin and DKK1 in alveolar bone and PDL, correlating with bone loss severity [37, 38] (Figure 2).

### 2.3 Wnt Activity Across Periodontal Compartments and Cell Types

The behavior of the Wnt signaling pathway within the periodontium is highly compartmentalized and cell‐type specific. In the PDL, mechanical tension such as that exerted by orthodontic forces activates canonical Wnt signaling in fibroblasts and progenitor cells, thereby driving both osteogenic and cementogenic differentiation [39, 40, 41]. By contrast, exposure of the PDL to compressive loads or inflammatory stress markedly upregulates several Wnt antagonists, including sclerostin, DKK1, and sFRPs, which serve to suppress new tissue formation and instead promote resorptive processes [42, 43, 44, 45].

A similarly nuanced regulatory role for Wnt signaling exists within cementum. Acute Wnt pathway stimulation in cementoblasts enhances cementogenic gene expression, including genes such as CEMP1 and CAP, and promotes matrix deposition [46, 47, 48]. However, persistent Wnt activation, as seen in sclerostin (SOST) knockout mice, does not result in increased cementum thickness and may actually impair cementocyte function [36]. This highlights the necessity for a tightly controlled Wnt signaling environment within the cementum, a process likely governed by additional epigenetic regulators [49, 50, 51].

### 2.4 Wnt Signaling and Mechanotransduction

Physiological mechanical forces are sensed by osteocytes and PDL fibroblasts via a specialized apparatus including integrins [27], Piezo1 ion channels [42], and primary cilia [52, 53]. In the periodontium, this sensing triggers a conserved anabolic cascade: loading downregulates Wnt antagonists (e.g., sclerostin) [29, 44] and stabilizes β‐catenin. This leads to the proliferation and osteogenic differentiation of Wnt‐responsive progenitors (e.g., Axin2+ cells), directly coupling force sensing to new bone formation at sites of tension [28, 54]. This process is synergized by the release of other osteogenic factors such as IGF‐1 and BMPs [40, 55, 56].

**FIGURE 3:**
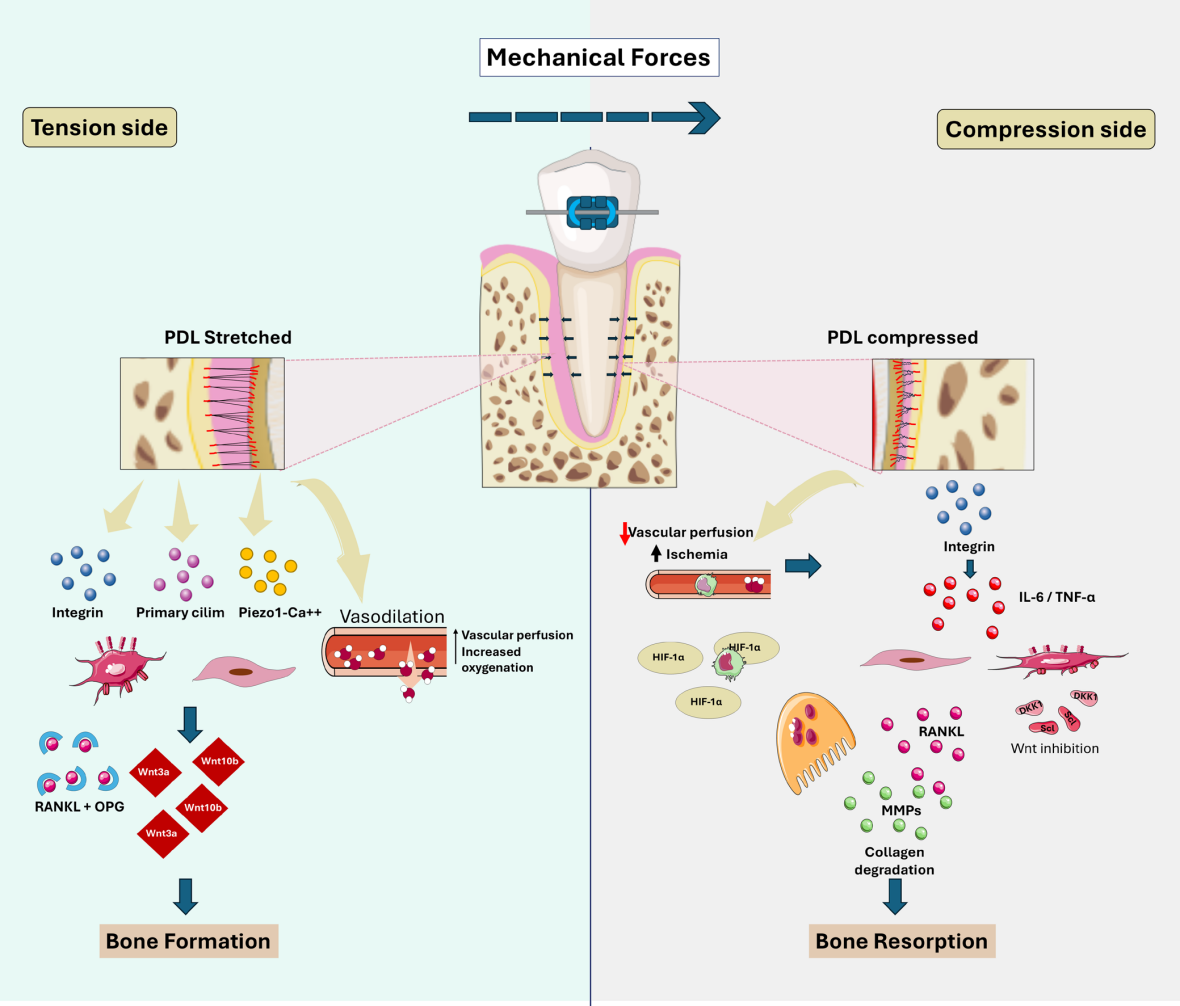
Mechanotransduction pathways in alveolar bone remodeling in an orthodontic tooth movement (OTM) model. Mechanical loading from occlusion or orthodontics creates strain and fluid shear stress that osteocytes detect through integrin signaling and primary cilia deflection, activating calcium and Wnt pathways. (A) In tension zones, PDL stretching triggers Piezo1/2 channel activation, improving blood flow and oxygen levels. This mechanical stimulation boosts Wnt/β‐catenin signaling through Wnt3a/10b, stimulating bone formation while inhibiting bone resorption through RANKL suppression. (B) In compression zones, blood vessel squeezing causes hypoxia that stabilizes HIF‐1α, increasing TNF‐α and IL‐6, RANKL, and decreasing OPG production. Osteocytes release Wnt blockers (DKK1, sclerostin), allowing osteoclast development. This spatial control of Wnt signaling—activated in tension areas and blocked in compression areas—coordinates tooth movement while protecting the surrounding bone structure.

This model provides a compelling, integrated view of periodontal mechanobiology. However, a significant portion of this cascade is inferred from analogous pathways in long bone osteocytes. Direct, real‐time visualization of force‐induced β‐catenin stabilization and progenitor cell fate specification within the living periodontium is lacking. Furthermore, the relative contributions of integrin, Piezo1, and ciliary signaling in initiating this cascade in different periodontal cell types (osteocytes vs. PDL fibroblasts) are not yet resolved, representing a key knowledge gap for targeted therapeutic modulation (Figure 3).

### 2.5 Pathological Disruption of Regenerative Wnt Signaling

#### 2.5.1 Mechanical Overload

When mechanical stress becomes excessive or chronic (e.g., occlusal trauma, poorly controlled orthodontic forces), the adaptive Wnt response is subverted. Overload induces hypoxia (HIF‐1α stabilization) [57, 58] and upregulates Wnt antagonists like sclerostin and DKK1 [44, 45, 59], actively suppressing canonical signaling. This shifts the RANKL/OPG balance toward resorption, uncoupling bone turnover and leading to clinical outcomes such as widened PDL spaces and progressive alveolar bone loss [13].

#### 2.5.2 Inflammatory Suppression (e.g., Periodontitis)

Chronic inflammation represents a potent and clinically significant disruptor. Inflammatory cytokines (TNF‐α, IL‐6) drive a marked upregulation of Wnt antagonists, including sclerostin, DKK1, and sFRP1, within both the PDL and alveolar bone [38]. The severity of this upregulation in human periodontitis correlates directly with clinical attachment loss and radiographic bone loss [38]. Critically, this pathway is therapeutically targetable: genetic deletion or antibody‐mediated neutralization of Sost rescues alveolar bone loss in experimental periodontitis, including in challenging conditions like type 2 diabetes [21, 33], establishing a definitive proof‐of‐principle.

#### 2.5.3 Pathway Crosstalk and Complexity

Pathological breakdown is exacerbated by signaling crosstalk. For instance, NF‐κB activation in inflammatory contexts (e.g., apical periodontitis) actively suppresses the Wnt/β‐catenin cascade [60]. Regenerative feedback mechanisms exist, such as exosomes from gingival mesenchymal stem cells that can deliver signals inhibiting NF‐κB while concurrently activating Wnt signaling [61]. Non‐canonical pathways also modulate the disease process, with elevated Wnt‐5a in gingival crevicular fluid correlating with inflammatory and osteoclastogenic activity in periodontitis [62].

#### 2.5.4 Implications for Peri‐Implantitis

The pathological principles of inflammatory Wnt suppression extend to the bone surrounding dental implants, where the dysregulated host‐microbiome interface is a key driver of disease [63]. Peri‐implantitis, a biofilm‐associated inflammatory disease leading to implant‐supporting bone loss, shares etiological features with periodontitis but is characterized by a more aggressive inflammatory response and rapid bone destruction.

Emerging molecular profiling reveals that the peri‐implant tissue microenvironment is fundamentally altered. RNA‐seq analyses demonstrate significant age‐related dysregulation in key pathways, including depressed Wnt signaling alongside heightened immune and inflammatory activity in aged mice compared to young controls [64]. In human disease, this manifests as significant immune dysregulation, including distinct macrophage polarization profiles that contribute to a pro‐inflammatory, pro‐osteoclastogenic state in peri‐implantitis lesions [65].

Within this pathological milieu, it is highly plausible that the potent upregulation of Wnt antagonists (e.g., sclerostin, DKK1) observed in periodontitis also occurs, actively suppressing the osteogenic repair necessary to maintain the bone‐implant interface. This represents a critical translational frontier. While direct evidence linking specific Wnt antagonist levels to peri‐implantitis progression is still accruing, proof‐of‐concept exists: sclerostin antibody treatment can enhance osseointegration in compromised bone [20]. The central, clinically significant question remains whether therapeutic local Wnt pathway activation can subvert the inflammatory suppression of bone formation and stabilize bone around ailing implants—a hypothesis now supported by mechanistic extrapolation and early molecular profiling [63, 64, 65], but awaiting targeted interventional validation.

### 2.6 Alveolar Bone Regeneration After Tooth Extraction

The specific role of Wnt signaling in extraction socket healing, a process of intramembranous ossification distinct from long bone fracture repair, remains an active area of investigation with limited direct evidence [66]. Preliminary findings suggest involvement: periodontal ligament stem cells (PDLSCs) contribute to socket healing [66], and local Wnt pathway modulation (e.g., sclerostin inhibition) can enhance bone formation in animal models, particularly in aged or estrogen‐deficient states [15, 19]. However, stem cell behavior is complex and context‐dependent. While key progenitor populations (Gli1+, Lepr+) are present [67, 68, 69, 70], they can adopt pathogenic states; for example, Lepr+ stromal cells in periodontitis can attenuate repair via CCRL2‐mediated Wnt inhibition [70]. Dedicated research is needed to clarify the precise regulation of Wnt‐responsive stem cells during alveolar socket regeneration (Table 3).

**TABLE 3:** Key Wnt antagonists and agonists in oral biology.

Molecule	Cell source	Role in periodontium	Effect of aging	Therapeutic modulation
Sclerostin	Osteocytes, some PDL cells	Inhibits Wnt/β‐catenin; suppresses bone formation	Upregulated	Antibody (Scl‐Ab) neutralization rescues bone loss
DKK1	Osteocytes, fibroblasts	Inhibits Wnt signaling	Upregulated	Dual antibody under development
Wnt3a, Wnt10b	Osteoblast‐lineage cells	Activates canonical pathway	Diminished expression	Recombinant protein (experimental)
Wnt5a, Wnt11	Multiple (inflammatory cells, fibroblasts)	Modulates non‐canonical signaling, inflammation, cementum repair	Increased in disease	Potential small‐molecule modulators
sFRP1	Fibroblasts, bone cells	Downregulates canonical pathway	Upregulated	Not clinically targeted

*Note:* Overview of major Wnt signaling molecules implicated in periodontal bone biology, including their cellular sources, roles within the periodontium, changes associated with aging, and current or potential avenues for therapeutic modulation.

## 3 Wnt Signaling and Aging

**FIGURE 4:**
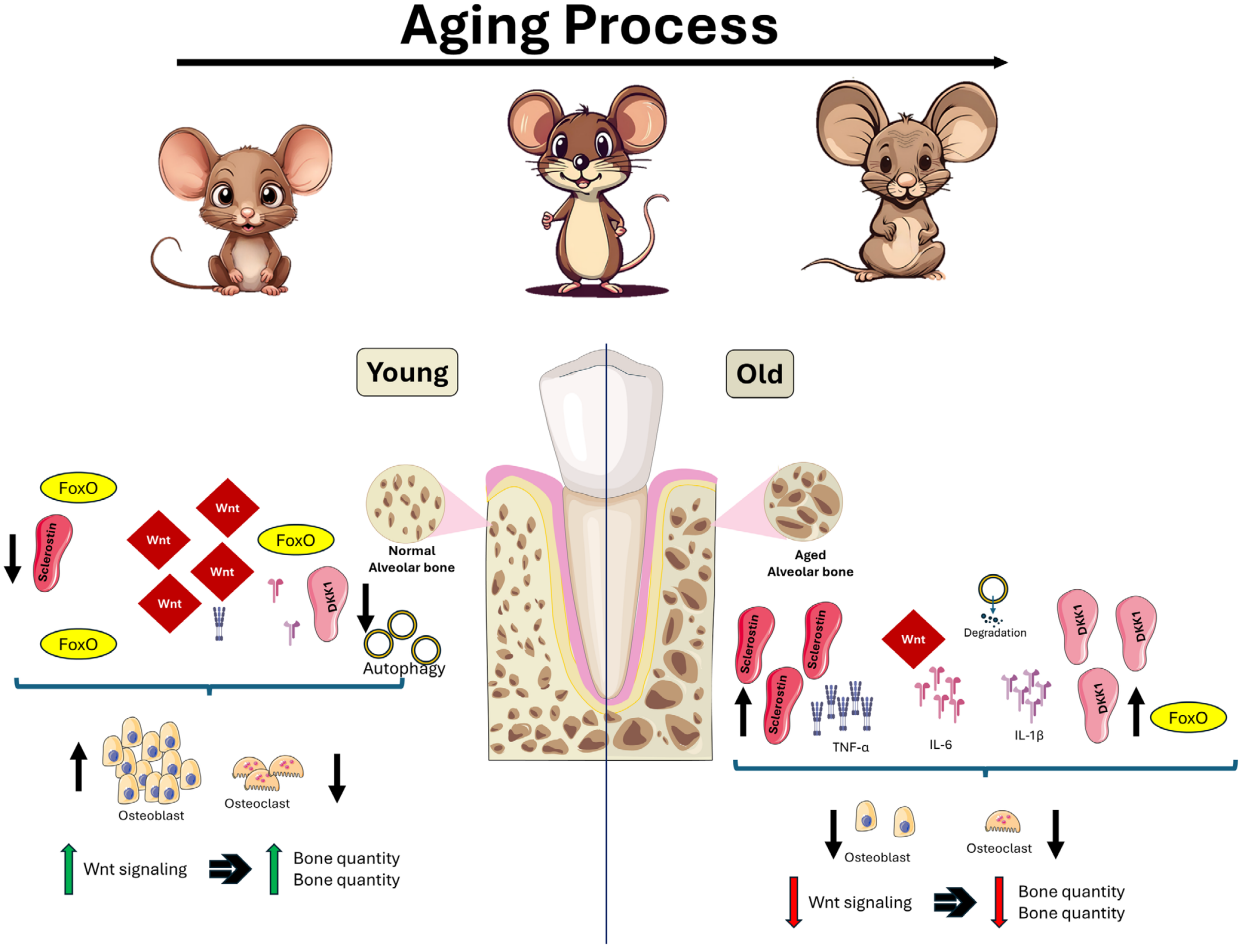
Age‐related dysregulation of alveolar bone homeostasis. The left panel depicts a healthy, young alveolar bone unit characterized by robust, homeostatic remodeling. Active osteoblasts, supported by functional osteocytes and efficient autophagy, respond to anabolic signals including potent canonical Wnt/β‐catenin activity. The right panel illustrates the aged alveolar bone microenvironment, where regenerative capacity is suppressed by a convergence of mechanisms. These include osteoblast dysfunction, cellular senescence with a pro‐inflammatory secretory phenotype (elevating TNF‐α, IL‐6, IL‐1β), and the potent inhibition of Wnt signaling via upregulated antagonists (sclerostin, DKK1), oxidative stress‐induced diversion of β‐catenin to FoxO transcription factors. Concomitant declines in autophagic clearance further exacerbate cellular stress. Collectively, these changes shift the RANKL/OPG balance, promoting osteoclast activity and leading to net alveolar bone loss.

Aging is a dynamic driver of tissue dysfunction, actively altering the responsiveness of cells within the components of the periodontium. The adaptability of these tissues depends on the coordinated activity of Wnt signaling networks, which respond to mechanical forces and inflammatory challenges [4, 35]. However, with advancing age, this regulatory machinery is progressively compromised. This manifests as impaired mechanotransduction [6], increased expression of Wnt antagonists like sclerostin [29, 71], and an exacerbated inflammatory response that further suppresses regenerative signaling [14, 38]. Below, we clarify mechanisms established through direct studies of the periodontium and those extrapolated from research in other bones (primarily long bone models), highlighting the similarities and unique features pertinent to oral health (Figure 4).

### 3.1 Canonical Wnt/β‐Catenin Signaling in Tissue Homeostasis and Aging

The principles of Wnt‐mediated regeneration, while promising, must be considered within the context of the systemic biological decline that accompanies aging [4]. In young, healthy tissues, the canonical Wnt/β‐catenin pathway orchestrates effective regeneration and repair. However, with advancing age, this same signaling pathway becomes fundamentally dysregulated.

Much of our understanding of age‐related impairment in Wnt signaling comes from research on long bones such as the femur and tibia. In these tissues, aging is known to disrupt skeletal homeostasis largely through changes in the canonical Wnt/β‐catenin signaling pathway. For instance, mechanical loading robustly activates Wnt signaling and promotes bone formation in younger individuals. As bones age, however, their mechanotransduction capacity diminishes [6, 9]. This reduced responsiveness is attributed primarily to increased secretion of Wnt antagonists, namely sclerostin and DKK1, by osteocytes, which actively suppress the osteogenic Wnt signal [7, 45, 71]. In addition to altered signaling, aged bone marrow stromal cells shift toward adipogenesis rather than osteogenesis, a transition driven by declining β‐catenin activity [7, 10]. Collectively, these changes create a systemic environment characterized by reduced anabolic capacity and increased skeletal fragility.

Recent studies demonstrate that many of these mechanisms are mirrored in oral tissues, providing direct evidence that the long bone findings are applicable within a periodontal context. For example, similar to what is observed in long bones, aged alveolar bone within the periodontium exhibits impaired activation of Wnt signaling in response to mechanical loading [6]. This deficit correlates with a failure to properly downregulate sclerostin, resulting in insufficient bone regeneration. Moreover, the expression of sclerostin in aged periodontal tissues is significantly elevated, paralleling the pattern seen in aging long bones [29, 71]. Systemic increases in sclerostin further contribute to bone loss in both skeletal sites, highlighting a shared endocrine‐like mechanism of age‐related bone degeneration [71].

Adding to this complexity, aging exacerbates inflammatory and resorptive responses in the periodontium [14], fostering a local environment that further antagonizes regenerative Wnt signaling—an effect similar to that observed in chronic inflammatory conditions affecting bone [38]. Additionally, recent discoveries have identified dysfunctional stem cell populations, such as pathogenic Lepr+ stromal cells, within aged periodontal tissues. These cells disrupt Wnt signaling and impede tissue repair, providing a cellular basis for the impaired regeneration seen in the aging periodontium [70].

### 3.2 Non‐Canonical Wnt Signaling (PCP and Ca^2+^ Pathways) in Aging Tissues

Non‐canonical Wnt ligands (e.g., Wnt5a, Wnt11) are crucial regulators of cell migration, tissue polarity, and inflammatory responses. Their activity becomes particularly significant in the dysfunctional cellular environment of aged bone, which is characterized by altered osteoblast/osteoclast activity and increased porosity [72, 73]. In this context, inflammatory cytokines like TNF‐α can elevate non‐canonical signaling. Wnt5a plays a dual role: it is essential for coordinating osteoclast–osteoblast coupling and maintaining bone formation [74], yet its dysregulation is also implicated in age‐related stem cell dysfunction [75]. This establishes a core principle from systemic biology: precise regulation of non‐canonical Wnt pathways is critical for skeletal homeostasis, and its disruption is a hallmark of aging.

Translating these mechanisms to oral tissues, emerging evidence confirms that non‐canonical Wnt signaling is relevant in periodontal aging and pathology. Chronic inflammation from periodontitis elevates WNT‐5a levels in gingival crevicular fluid, paralleling increased local inflammation and osteoclast activity [62]. Inflammatory upregulation of Wnt5a can block cementogenic differentiation of periodontal cells, further hindering repair. Interestingly, certain non‐canonical Wnt pathways might also offer protection; for example, Wnt4 can inhibit NF‐κB–mediated inflammation in bone, raising the possibility of similar benefits in oral tissues.

Yet, many questions remain unanswered. What roles do ligands like Wnt11 and Wnt16 play in the aging of alveolar bone, PDL, and cementum? How do mechanical forces, which are a defining feature of the oral environment, modulate PCP and Ca^2+^ pathways in aged tissues? And is the protective effect of Wnt4 observed in long bones also present in oral tissues? Addressing these gaps will be essential for developing therapies that harness non‐canonical Wnt signaling to preserve periodontal health in an aging population.

### 3.3 Crosstalk With Forkhead Box O (FoxO) Transcription Factors

#### 3.3.1 Integrating Metabolism and Oxidative Stress Response in Aging

FoxO transcription factors play a central role in balancing cellular stress resistance and regenerative ability, an equilibrium that becomes increasingly disrupted with age. In long bones, rising oxidative stress is a major cause of bone loss [76]. Under these conditions, β‐catenin shifts from its canonical role with TCF/LEF to partnering with FoxO proteins, prioritizing antioxidant defenses at the expense of bone‐forming gene programs [76]. Though this adaptation initially protects cells, chronic FoxO activation ultimately suppresses Wnt signaling and slows bone formation [77]. This effect occurs via mechanisms like β‐catenin sequestration [78] and CBP‐mediated acetylation of FOXO4 [79]. Over time, FoxO overactivity contributes to age‐related bone decline—a process independent of, but worsened by, sex steroid deficiency [80]. Thus, in long bones, the drive for stress survival directly undermines tissue regeneration.

In oral tissues, evidence for FoxO–Wnt crosstalk is just beginning to surface. The periodontium's unique microenvironment, marked by constant inflammatory and metabolic stress from the oral microbiome [14], suggests this signaling axis could be highly relevant locally. Systemic factors may also influence outcomes; for example, parathyroid hormone (PTH) can lower oxidative stress and promote bone formation in aging long bones [81], hinting at ways to restore FoxO–Wnt balance. Early studies show that the FoxO–Wnt interaction shapes the trade‐off between resilience and regeneration in oral tissues, with FoxO1 playing varying roles in young and aged models [82].

However, important questions remain. What specific functions do FoxO isoforms (FoxO1, 3a, 4) have in periodontal cells such as osteocytes, ligament stem cells, and cementoblasts? Can anabolic therapies like PTH or Sclerostin antibody also modulate the FoxO–Wnt axis in the periodontium? How do broader aging pathways—such as declines in sirtuin 1 (Sirt1) activity [78] affect FoxO–Wnt signaling in oral tissues?

Clarifying these mechanisms is crucial for future therapies. Strategies such as modulating FoxO acetylation or combining FoxO‐targeted approaches with other bone anabolic agents may help rebalance this signaling axis, ultimately enhancing resilience and regeneration of alveolar bone and periodontal tissues as we age.

### 3.4 Wnt Signaling, Cellular Senescence, and Senescence‐Associated Inflammation

Research on long bones has established that the accumulation of senescent cells is a hallmark of skeletal aging and a major contributor to osteoporosis [83, 84]. Senescent osteocytes and bone marrow stromal cells develop a robust senescence‐associated secretory phenotype (SASP), which fosters a pro‐inflammatory and tissue‐destructive microenvironment, ultimately weakening bone structure and increasing fragility [85]. Central to this process is the interplay with Wnt signaling: a decline in pro‐regenerative Wnt ligands such as Wnt3a is required for mesenchymal stem cells to enter senescence [86], while an increase in certain Wnts, notably Wnt16, can drive osteoblasts toward senescence [87]. Thus, there is a bidirectional relationship: Wnt pathway activity governs cellular senescence, and senescent cells, through their SASP, secrete factors that further suppress Wnt signaling. Interventions targeting this axis, whether by clearing senescent cells (senolytics) [88], or modulating oxidative stress with agents like Astaxanthin or Pyrroloquinoline Quinone [89, 90], have shown efficacy in mitigating bone loss in aging models, validating this pathway as a therapeutic target in systemic skeletal aging.

Emerging research indicates that this senescence‐inflammation–Wnt suppression cycle is also present in oral tissues, but with unique features tied to the periodontal environment. Periodontitis‐induced chronic inflammation exposes cells to continuously high levels of cytokines such as TNF‐α, IL‐1β, and IL‐6, which intensify inflammatory responses and promote alveolar bone resorption [14]. This persistent inflammatory milieu favors cellular senescence in the periodontium. Experimental studies parallel findings from long bones: inflamed human periodontal tissues exhibit elevated levels of the Wnt antagonists DKK1 and sclerostin, and these correlate with the severity of clinical bone loss [38]. Importantly, inhibition of sclerostin either genetically or using antibodies can rescue alveolar bone in animal models of periodontitis [33, 38], confirming the pathogenic role of Wnt suppression. Additionally, non‐canonical Wnt signaling contributes to oral tissue inflammation; for example, Wnt4 can dampen bone inflammation by inhibiting NF‐κ [91], while its disruption is linked to occlusal trauma [92]. Elevated Wnt5a is also associated specifically with inflammatory periodontitis.

While the “Senescence → SASP → Wnt Suppression → Impaired Regeneration” cycle is well‐defined in long bone biology, the oral cavity is exposed to constant microbial and mechanical stresses, likely accelerating both senescence and Wnt pathway inhibition. The unique oral microenvironment, shaped by polymicrobial biofilm and dynamic mechanical loading, may hasten the progression of this pathogenic cycle, making it particularly challenging for periodontal tissues to maintain regenerative capacity. Thus, therapeutically targeting this multiple‐hit pathway, whether through senolytics [88], inhibition of Wnt antagonists such as sclerostin [21, 33], or activation of protective Wnt signaling (e.g., Wnt4) [91], holds strong promise for rejuvenating the aged and inflamed periodontium.

Despite these advances, critical questions remain unanswered. How do different subtypes of senolytics interact with Wnt signaling within distinct periodontal niches? What roles do non‐canonical Wnt ligands play in modulating or mitigating senescence‐induced inflammation in the periodontium? Can senolytic or Wnt‐based therapies developed for long bone disease be adapted to the unique microenvironmental challenges of the oral cavity, and if so, how do we optimize their efficacy?

### 3.5 Autophagy, Mitochondrial Dysfunction, and Wnt Signaling in Aging

Long bone studies offer crucial insights into the cellular mechanisms underlying bone maintenance and aging. Robust autophagy and mitochondrial function within osteoblast lineage cells are essential for preserving bone mass and strength. Elevating autophagy, such as through genetic upregulation of TFEB—results in increased bone mass [93, 94], whereas suppressing autophagy in osteocytes causes a skeletal phenotype that mimics aging [95, 96]. Mitochondrial health is equally important; oxidative stress or reduced autophagy in osteoblasts impairs the bone's ability to respond to mechanical forces, recapitulating age‐related decline [97]. Furthermore, in disease contexts, autophagy can negatively regulate Wnt signaling and its suppression can drive inflammatory bone resorption [98]. Mitochondrial dysfunction is also a key mediator in systemic diseases such as Leigh syndrome, contributing to osteoporosis and fragile bone [99, 100]. These findings establish autophagy and mitochondrial health as key, cell‐intrinsic requirements for bone formation, adaptation, and resistance to inflammatory and metabolic stress.

Recent research indicates that similar cellular pathways are relevant in oral tissues but manifest in distinct, site‐specific ways. In the periodontium, diabetes is known to worsen periodontitis by promoting mitochondrial dysfunction [101], and infection with *P. gingivalis* directly triggers mitochondrial fragmentation and dysfunction in host cells [102]. Unlike long bones, the oral environment's unique exposure to microbial biofilms and frequent inflammation means impaired autophagy and mitochondrial failure are likely common and may converge to suppress regenerative Wnt signaling, compromising alveolar bone and PDL maintenance. Studies also show that mitochondrial quality governs the differentiation of dental stem cells, and its failure potentially impairs oral tissue repair [99]. Non‐canonical crosstalk has been observed where autophagy and mitochondrial dysfunction interact with inflammatory and Wnt pathways, further aggravating periodontal breakdown [98, 101].

Given the parallels seen in cellular stress responses, the foundational knowledge from long bone biology strongly informs hypotheses for oral tissues. However, direct studies in the periodontium remain limited, and major questions persist: How does autophagic flux specifically affect Wnt signaling in human periodontal ligament stem cells or cementoblasts? Can interventions that activate TFEB or protect mitochondria rescue periodontal cell function under inflammatory conditions? Would regimens that combine a Wnt activator (like sclerostin antibody) with an autophagy inducer offer synergistic benefits for periodontal regeneration?

### 3.6 Mechanosensing Dysfunction

#### 3.6.1 Impaired Wnt Response in the Aged Periodontal Complex

Mechanotransduction, the conversion of mechanical forces into cellular signals, is essential for maintaining bone integrity throughout life. Pivotal research in long bones has demonstrated that aging impairs mechanotransduction by diminishing the activation of Wnt signaling in response to mechanical loading [6] and reducing the transcriptional responsiveness of bone cells to force [9]. As a result, these deficits are recognized as key contributors to age‐related systemic bone loss [71], setting a precedent for the study of mechanotransduction in other tissues, such as the periodontium.

Building on lessons from skeletal biology, direct research in oral tissues has identified PIEZO1 as a principal mechanosensor in periodontal health and disease. PIEZO1 is necessary for force‐driven alveolar bone remodeling via Wnt signaling in PDL cells [42], supports tension‐induced osteogenic differentiation in PDL stem cells [103], and controls the balance between proliferation and mineralization in human cementoblasts [104]. Experimental findings also show that activating PIEZO1 can safeguard against periodontitis exacerbated by traumatic occlusion [105]. Remarkably, PIEZO1's impact varies by cell type and context: in osteoclasts, it protects against inflammatory bone loss through a mechanism independent of Ca^2+^ signaling [106]. Collectively, these observations establish PIEZO1 as a pivotal regulator of adaptation, remodeling, and inflammation in periodontal tissues.

While the principle that mechanosensing capacity declines with age is established in long bones, it remains unclear how aging affects PIEZO1‐mediated mechanotransduction in the periodontium. Does PIEZO1's expression, sensitivity, or cell‐type specific protective functions decrease with age, potentially contributing to periodontal fragility? Is the anti‐inflammatory, bone‐protective role of osteoclastic PIEZO1 compromised in older individuals, accelerating bone loss in periodontitis? Does anabolic signaling through PIEZO1 in PDL stem cells become uncoupled as tissues age? The constant mechanical and inflammatory stresses unique to the oral cavity may place exceptional demands on this network, making its age‐related degradation a probable factor in periodontal breakdown.

### 3.7 Systemic Influences

#### 3.7.1 Sex Hormones and Their Interaction With Wnt in Aging

Sex hormones, such as estrogen, are essential for maintaining bone health, largely by regulating the Wnt signaling pathway. In long bones, these hormones promote bone formation and strength through several coordinated mechanisms. Estrogen increases Wnt activity in osteoblasts and suppresses Wnt inhibitors like sclerostin, supporting bone‐building processes [107]. Crucially, estrogen receptors and Wnt/β‐catenin signaling work together to encourage osteogenic differentiation of mesenchymal progenitor cells [107]. Androgens also boost Wnt activity, further enhancing the environment for bone growth. This synergy between sex hormones and Wnt signaling is a cornerstone of skeletal endocrinology.

As individuals age, hormone levels, especially estrogen, decline, most notably after menopause. This hormonal shift disrupts the balance with Wnt signaling, leading to weaker bone formation and increased bone resorption [71, 108]. As a result, bone metabolism tips toward loss, making osteoporosis more likely. Long bone research has defined the molecular details: reduced estrogen not only diminishes Wnt signaling but also increases expression of Wnt antagonists such as sclerostin and DKK1, which suppress the activity of osteoblasts and contribute to bone mass erosion [71]. Notably, some Wnt‐mediated protective effects, particularly those involving Wnt16, remain active even without estrogen [109], highlighting the complexity of the regulatory system. Furthermore, reduced estrogen impairs the bone's ability to activate Wnt signaling in response to mechanical loading [108]. Therapeutically, targeting these molecular interactions has shown promise. For example, using antibodies to block sclerostin can restore Wnt pathway activity and reverse hormone‐related bone loss in animal models and clinical trials, confirming that Wnt activation can effectively compensate for declining sex hormones [110, 111, 112].

Recent research provides direct evidence for these hormonal and molecular mechanisms in the oral cavity. In rodent models simulating menopause through ovariectomy, estrogen deficiency sharply accelerates alveolar bone loss by suppressing Wnt pathway activity and promoting bone resorption [113, 114]. This effect is compounded by increased production of local Wnt antagonists and pro‐resorptive cytokines [14], mirroring the mechanisms seen in long bones. Importantly, targeted therapies that modulate Wnt signaling have been shown to be effective in the oral context: for example, antibody‐mediated inhibition of sclerostin corrects alveolar bone loss in models of ovariectomized rats or type 2 diabetic periodontitis [21, 115] and enhances socket repair after estrogen withdrawal in rats [19]. These findings directly affirm that reversing Wnt suppression can support oral bone regeneration in hormonally compromised situations. Additionally, systemic rises in sclerostin remain associated with age‐related and estrogen withdrawal‐related bone loss in the periodontium [71].

Despite strong mechanistic parallels, emerging evidence confirms that the molecular landscape in the craniofacial bone is distinct from that of long bones. Site‐specific differences in osteoblast phenotype, mechanical loading response, and estrogen receptor‐related gene expression have been documented [116]. These insights show the need for continued oral‐specific research, rather than simple extrapolation from skeletal models, to define the precise regulation of Wnt and related pathways in the aging craniofacial bone.

## 4 Wnt‐Targeted Therapeutic Strategies for Alveolar Bone Regeneration

The Wnt/β‐catenin signaling pathway is essential for skeletal homeostasis and tissue regeneration. Its dysregulation drives age‐related bone loss, making it a prime target for intervention in both systemic osteoporosis and oral bone health. Therapeutic strategies now range from FDA‐approved systemic approaches to innovative, experimental treatments focused on localized oral regeneration.

### 4.1 FDA‐Approved Systemic Therapy

Sclerostin antibody (SclAb), clinically known as Romosozumab, is a monoclonal antibody developed to neutralize sclerostin, a potent and secreted inhibitor of canonical Wnt signaling, a central regulator of bone formation [4]. Sclerostin, produced by osteocytes, binds LRP5/6 co‐receptors on osteoblasts and blocks Wnt ligands from activating the signaling cascade required for β‐catenin stabilization and osteogenic gene expression [4, 29]. This action provides a physiological brake on bone mass formation. However, in aging and chronic inflammation, sclerostin levels rise, systemically [71] and locally in inflamed periodontal tissues [28], contributing to suppressed bone regeneration. SclAb restores Wnt activation, re‐engaging osteoblast function, stimulating bone formation, and suppressing resorption.

Clinically, Romosozumab is administered monthly via subcutaneous injection and is FDA‐approved for up to 12 months in postmenopausal women at high fracture risk. It rapidly increases bone formation, reduces resorption, and achieves significant gains in bone mineral density, with up to 73% reduction in vertebral fracture risk compared to placebo [112].

The therapeutic potential of sclerostin inhibition extends to diverse models of bone disease and oral pathology. In rodent models of estrogen deficiency, SclAb restores alveolar bone mass and improves healing post‐extraction [19]. In periodontitis models, both genetic deletion and antibody blockade of sclerostin rescue alveolar bone loss [17, 33]. In type 2 diabetic periodontitis, SclAb reverses severe bone loss [21]. SclAb also enhances alveolar bone quality and dental implant osseointegration in conditions such as osteoporosis, osteogenesis imperfecta, and X‐linked hypophosphatemia [18, 20, 115, 117, 118], demonstrating its capacity to overcome multiple barriers to oral bone regeneration.

Studies using systemic administration of SclAb have reported increased bone mass even in wild‐type mice, indicating that SclAb therapy has potential for bone augmentation in healthy individuals [20, 21, 115]. Preclinical models show that local administration of SclAb in alveolar bone defects leads to significant enhancement of bone formation when delivered at sufficiently high concentrations, resulting in marked increases in bone volume and trabecular thickness. This targeted delivery method enables focused bone regeneration at specific defect sites and may help limit systemic side effects. However, the benefits are dose‐dependent, as lower‐dose local formulations have not provided meaningful improvements compared to controls [117, 119].

Despite its anabolic benefits, systemic romosozumab carries a black‐box warning for increased major adverse cardiovascular events (MACE), as shown in the ARCH trial [120]. Sclerostin serves a protective role in vascular health, and its inhibition may promote arterial calcification and proliferation, raising cardiovascular risk [121, 122]. Consequently, systemic therapy is restricted to 1 year and only prescribed for patients without cardiovascular risk factors.

The evidence for local efficacy, coupled with systemic safety limitations, strongly supports the need for advanced localized SclAb delivery systems in oral medicine. However, it is important to note that local delivery has been assessed exclusively in preclinical animal studies; no clinical trials in humans have yet tested this approach. A significant challenge remains: the pharmacokinetics of locally administered SclAb, including its absorption, retention, and release profile within the oral environment, have not been fully characterized even in these animal studies. Technical obstacles such as rapid salivary clearance, masticatory forces, and microbial degradation further complicate sustained and controlled drug availability. Future progress will rely on engineered biomaterials that preserve biologic integrity and ensure precise, site‐specific activation. Combination protocols with anti‐inflammatories or senolytics may further improve outcomes, especially in complex or aged tissues. Ultimately, the goal is to deliver potent local bone regeneration, as established in preclinical models, while thoroughly understanding and optimizing local pharmacokinetics to maximize efficacy and minimize risk—before considering human clinical application (Table 4).

**TABLE 4:** Comparative summary of modalities for alveolar bone regeneration.

Modality	Delivery route	Clinical evidence	Efficacy in aging models	Advantages	Limitations/risks
Sclerostin Ab (Romosozumab)	Systemic/subcutaneous	FDA‐approved (osteoporosis), preclinical oral	Proven in rodents (aged, estrogen‐def.)	Potent anabolic, robust rescue	Cardiovascular risk, need for local delivery
Local Sclerostin Ab	Local/injectable/graft	Preclinical only	Effective in aged rodents	Enhanced targeting, reduced systemic risk	Uncharacterized PK/delivery in oral tissues
DKK1 Ab	Systemic/experimental	Early stage	Potent in some models	Synergistic with Scl‐Ab	No safety profile yet
BMP‐2	Local/scaffold	FDA‐approved (craniofacial)	Safe, effective	Well‐established, osteoinductive	Limited efficacy in severe Wnt suppression, cost
EMD	Local/graft	FDA‐cleared	Effective, long‐term studies	Soft tissue and bone healing	Less effective in severe bone loss
MSC/exosome therapy	Local/scaffold	Preclinical	Promising	Immunomodulatory, less invasive	Experimental, unclear long‐term outcomes

*Note:* Comparison of current and emerging therapeutic modalities for alveolar bone regeneration, detailing delivery routes, level of supporting clinical or preclinical evidence (including efficacy in aged models), main advantages, limitations and risks. This table facilitates an at‐a‐glance evaluation of the translational and clinical readiness of each approach.

### 4.2 Experimental Wnt Therapies

#### 4.2.1 Dual Inhibitor Antibodies and Wnt Agonists

Building on the therapeutic principle of Wnt pathway activation, research is moving beyond single‐target inhibition such as sclerostin or DKK1 alone to multi‐target strategies for greater efficacy. Notably, concurrent inhibition of both sclerostin and DKK1 in a rat model of alveolar bone loss more effectively preserves and augments bone volume compared to single‐target approaches [123]. This establishes direct proof that dual antagonism of Wnt inhibitors may be superior for oral bone regeneration. Along these lines, newly developed bispecific antibodies that neutralize both sclerostin and DKK1 have shown synergistic osteogenic effects in systemic skeletal models, improving bone mass and fracture repair [124]. Mechanistically, this approach releases the full osteoanabolic potential of DKK1 inhibition that would otherwise be reduced by compensatory upregulation of sclerostin [125]. DKK1 blockade also shows efficacy in genetic bone fragility, such as osteogenesis imperfecta [126], broadening its therapeutic promise. Together, these findings support continued development of bispecific agents for complex oral bone defects.

Direct Wnt pathway agonism through recombinant Wnt proteins is a complementary frontier. Here, lipid‐mediated stabilization technologies enable the delivery and efficacy of proteins such as Wnt3A for therapeutic use [127]. Liposomal Wnt3A (L‐Wnt3A) formulations enhance in vivo stability and allow for precise local application. Studies show that these protein therapies can rejuvenate bone‐forming capacity, even in autografts from aged animals [15], and accelerate healing in rodent injury models by activating Wnt‐responsive osteoprogenitors. Regenerative potential is further increased when combined with biomaterial scaffolds. However, these protein‐based strategies remain experimental; open questions remain regarding their durability in the oral environment, optimal biomaterial integration, and long‐term safety before clinical use.

Mesenchymal Stem Cell (MSC) Therapies, including exosome‐, scaffold‐, or cell‐based approaches, remain experimental for oral bone regeneration. Preclinical studies are promising, showing enhanced healing. Relevant to this review, exosomes derived from gingival MSCs have been shown to cross‐regulate Wnt and NF‐κB signaling in the periodontal inflammatory microenvironment [61], and direct application of a WNT protein therapeutic can improve bone formation in grafts from aged animals [15], providing proof‐of‐principle for Wnt‐mediated regeneration.

### 4.3 Comparisons to Current Clinical Modalities

Wnt‐targeted therapies must be considered alongside well‐established clinical modalities, particularly bone morphogenetic protein‐2 (BMP‐2) and enamel matrix derivative (EMD).

BMP‐2 is FDA‐approved for certain craniofacial indications including sinus augmentation and ridge preservation. Its osteoinductive activity is intimately linked to the Wnt pathway, as BMP‐2 facilitates osteoblast differentiation and mineralization through an autocrine Wnt loop [128]. Preclinically, BMP‐2 incorporated into biomimetic grafts promotes periodontal and alveolar regeneration in large animal models [129, 130], and randomized clinical trials confirm rhBMP‐2's efficacy in augmenting alveolar ridge volume in humans [131].

EMD is an FDA‐cleared biologic derived from porcine tooth buds and widely used for periodontal regeneration. EMD supports soft tissue healing and modulates inflammation [132], in part by inducing growth factor and Wnt target gene expression. Clinically, combining EMD with bone grafts treats severe intrabony defects effectively [133], and EMD combined with guided tissue regeneration yields predictable outcomes [134]. Long‐term studies indicate stable periodontal regeneration with EMD use over a decade [135].

Both BMP‐2 and EMD are valued for their safety, accessibility, and ability to engage the patient's endogenous Wnt pathway. However, their efficacy can be diminished in patients with severely suppressed Wnt signaling—a hallmark of advanced age, diabetes, or metabolic disease, where Wnt antagonists like sclerostin are elevated. In these contexts, direct Wnt pathway activation via sclerostin‐neutralizing antibodies has demonstrated robust preclinical efficacy in models of diabetic and osteoporotic periodontitis [19, 21]. Therefore, while BMP‐2 and EMD remain clinical mainstays, next‐generation Wnt‐targeted strategies are poised to overcome age‐related regenerative deficits, potentially enabling future combination or sequential therapeutic paradigms.

### 4.4 Future Perspectives and Research Directions in Wnt Signaling /Aging

Despite dramatic advances in our understanding of Wnt signaling and its role in skeletal aging, there remain substantial limitations and knowledge gaps when it comes to the craniofacial complex and oral tissues. Much of today's mechanistic insight about Wnt comes from long bone studies, but the periodontium—composed of the alveolar bone, PDL, and cementum—offers a more dynamic microenvironment, exposed to unique mechanical, inflammatory, and microbial stresses. Direct research focusing on Wnt signaling in these oral tissues is still in its infancy, making extrapolation from systemic skeletal models an interim solution with inherent shortcomings.

One key limitation is the incomplete mapping of Wnt‐responsive cell populations within the periodontium. While recent work has identified populations such as Gli1‐positive and Lepr‐positive cells, the full diversity of stem/progenitor groups, their fate decisions, and their responses to aging remain unresolved. Additionally, mechanosensing pathways—like those involving PIEZO1 channels and integrins—are increasingly recognized as pivotal in translating chewing forces into cellular regenerative signals via Wnt, yet how aging alters these sensors and their downstream effects is poorly understood in oral tissues compared to the femur or tibia. Does the aged periodontium lose its ability to “sense” and respond to force, and if so, by what cellular and molecular mechanisms?

Crosstalk with inflammatory pathways further complicates the landscape. Chronic oral inflammation, often driven by lifelong exposure to microbial biofilms, intensifies Wnt suppression via antagonists like sclerostin and DKK1. The precise molecular mechanisms by which inflammatory and senescent processes inhibit Wnt‐mediated regeneration in aged oral tissues are not fully delineated. Non‐canonical Wnt ligands (such as Wnt5a, Wnt11, and Wnt16) also play underexplored roles in modulating immune responses and cell polarity in the periodontium, presenting intriguing but poorly defined therapeutic targets.

Therapeutic translation encounters its own set of challenges. While systemic sclerostin antibody (romosozumab) is effective for osteoporosis, its cardiovascular risks limit long‐term use. Local delivery of Wnt‐targeted drugs in the oral cavity is promising but unproven: How do these agents behave in a saliva‐rich, mechanically active environment, and can they maintain stability and efficacy within aged tissue? Preclinical models suggest potent results, but clinical studies, especially in older human populations, are almost nonexistent.

Senescence—the progressive decline in cell renewal capacity—is another frontier where oral research lags behind skeletal biology. The “vicious cycle” of senescence‐associated inflammation promoting Wnt suppression (and vice versa) is established in long bones but remains largely hypothetical in the periodontium. Moreover, whether emerging interventions like senolytics or autophagy enhancers can rejuvenate aged periodontal tissue through Wnt pathway modulation is a tantalizing but unanswered question.

Finally, hormonal factors, particularly the decline of estrogen with age, are known to interact with Wnt signaling and accelerate bone loss. Rodent studies have shown parallels in oral bone, yet the nuances of hormone–Wnt interactions—and optimal therapies to counteract their decline—require much deeper exploration in clinical settings.

Looking ahead, the field faces an exciting challenge. There is a clear need for direct oral‐specific research, employing advanced lineage tracing, single‐cell profiling, and in vivo mechanotransduction studies in aged models. Novel drug delivery systems—perhaps using bioengineered scaffolds or hydrogels customized for periodontal tissues—must be developed and rigorously tested for efficacy and safety. Exploring combination therapies that integrate Wnt agonism, senolytic agents, autophagy enhancers, and hormone replacement could offer synergistic regenerative effects. Importantly, understanding how mechanical forces, chronic inflammation, and the oral microbiome interact with aging and Wnt signaling would allow for multi‐pronged interventions targeting the root causes of periodontal decline.

Moreover, unraveling the roles of non‐canonical Wnt ligands and mapping their specific effects across different cell types of the periodontium may reveal overlooked protective mechanisms or novel targets for therapy. Lastly, translating preclinical successes to human trials, especially those focused on older, medically complex patients, will be critical to achieve tangible improvements in oral regenerative medicine (Table 5).

**TABLE 5:** Knowledge gaps and future research directions in periodontal Wnt signaling.

Knowledge gap	Clinical relevance	Suggested research direction	Potential impact
Aging effect on PIEZO1 and mechanosensors	Explains failed adaptation in elders	In vivo imaging, single‐cell analysis in aging models	More effective mechano‐based therapies
Fate and function of Wnt‐responsive stem cells (Gli1+, Lepr+)	Targeting regeneration after injury/extraction	Lineage tracing, molecular profiling in aged/human tissues	Development of targeted regeneration strategies
Local vs. systemic pharmacokinetics of Wnt modulators	Safety, efficacy, side‐effects	PK/PD studies, biomaterial release optimization	Safer, targeted local therapies
Non‐canonical Wnt roles in inflammation, cementum repair	Precision therapies for periodontitis/peri‐implantitis	Direct tissue‐level mechanistic studies, new modulators	Better outcomes in oral inflammatory diseases
Effect of metabolic disease (diabetes) on Wnt therapy response	Higher risk population, poor outcomes	Integrated metabolic/Wnt modulation research	Broader therapy applicability
Combination therapy (Wnt agonism + senolytics/autophagy)	Addressing multi‐pathway aging	Preclinical studies in aged, complex models	Synergistic, robust regeneration

*Note:* Critical areas where knowledge remains limited regarding Wnt biology and therapeutic modulation in the periodontium, paired with the direct clinical relevance of each gap. Suggested future research directions propose methodologies to address these uncertainties and outline the potential impact on clinical practice and patient outcomes.

## 5 Conclusion

Aging in the periodontium leads to profound shifts in tissue dynamics, with Wnt signaling at the heart of maintaining bone and ligament integrity. As age‐related disruption of Wnt pathways drives periodontal degeneration, the consequences extend far beyond oral health; compromised periodontium exacerbates systemic diseases and worsens quality of life through pain, impaired function, and increased risk of tooth loss. Together, these insights reveal a strong clinical rationale for creating interventions that reactivate Wnt pathways and support periodontal regeneration in aging individuals. Innovative approaches targeting Wnt signaling offer promising solutions, providing not only the potential to preserve or restore dental support but also to improve overall health outcomes. Continued research focused on translating these findings into safe, effective clinical strategies is essential for enhancing well‐being and longevity in the aging population.

## Disclosure

This review manuscript was proofread for grammar and clarity using DeepSeek (OpenAI, v3.2). This tool also assisted in identifying potential gaps in citations; any missing references were subsequently reviewed and manually added by the authors. No content or scientific interpretations were generated by AI. All original text, synthesis, and conclusions are the work of the authors.

## Data Availability

No primary datasets were generated for this review. The manuscript synthesizes findings from the existing scientific literature, as referenced. All cited publications are accessible through their respective publishers or repositories.

## References

[1] S. Hung , The Periodontium, edited by L. Oav (Springer‐Verlag, 1986).

[2] E. Hlaing , Y. Ishihara , N. Odagaki , Z. Wang , M. Ikegame , and H. Kamioka , “The Expression and Regulation of Wnt1 in Tooth Movement‐Initiated Mechanotransduction,” American Journal of Orthodontics and Dentofacial Orthopedics 158, no. 6 (2020): e151–e160, 10.1016/j.ajodo.2020.08.006. 33139146

[3] A. Li , R. Z. Thomas , L. van der Sluis , G. H. Tjakkes , and D. E. Slot , “Definitions Used for a Healthy Periodontium—A Systematic Review,” International Journal of Dental Hygiene 18, no. 4 (2020): 327–343, 10.1111/idh.12438. 32330350 PMC7687205

[4] P. Duan and L. F. Bonewald , “The Role of the Wnt/Beta‐Catenin Signaling Pathway in Formation and Maintenance of Bone and Teeth,” International Journal of Biochemistry & Cell Biology 77 (2016): 23–29, 10.1016/j.biocel.2016.05.015. 27210503 PMC4958569

[5] N. Tokavanich , M. N. Wein , J. D. English , N. Ono , and W. Ono , “The Role of Wnt Signaling in Postnatal Tooth Root Development,” Frontiers in Dental Medicine 2 (2021): 769134, 10.3389/fdmed.2021.769134. 35782525 PMC9248717

[6] N. Holguin , M. D. Brodt , and M. J. Silva , “Activation of Wnt Signaling by Mechanical Loading Is Impaired in the Bone of Old Mice,” Journal of Bone and Mineral Research 31, no. 12 (2016): 2215–2226, 10.1002/jbmr.2900. 27357062 PMC5397287

[7] M. Rauner , W. Sipos , and P. Pietschmann , “Age‐Dependent Wnt Gene Expression in Bone and During the Course of Osteoblast Differentiation,” Age 30, no. 4 (2008): 273–282, 10.1007/s11357-008-9069-9. 19424851 PMC2585653

[8] Z. Zhong , N. J. Ethen , and B. O. Williams , “WNT Signaling in Bone Development and Homeostasis,” WIREs Developmental Biology 3, no. 6 (2014): 489–500, 10.1002/wdev.159. 25270716 PMC4199871

[9] C. J. Chermside‐Scabbo , T. L. Harris , M. D. Brodt , et al., “Old Mice Have Less Transcriptional Activation but Similar Periosteal Cell Proliferation Compared to Young‐Adult Mice in Response to In Vivo Mechanical Loading,” Journal of Bone and Mineral Research 35, no. 9 (2020): 1751–1764, 10.1002/jbmr.4031. 32311160 PMC7486279

[10] G. L. Galea , L. B. Meakin , M. A. Harris , et al., “Old Age and the Associated Impairment of Bones' Adaptation to Loading Are Associated With Transcriptomic Changes in Cellular Metabolism, Cell‐Matrix Interactions and the Cell Cycle,” Gene 599 (2017): 36–52, 10.1016/j.gene.2016.11.006. 27840164 PMC5139832

[11] S. W. McCormack , U. Witzel , P. J. Watson , M. J. Fagan , and F. Groning , “Inclusion of Periodontal Ligament Fibres in Mandibular Finite Element Models Leads to an Increase in Alveolar Bone Strains,” PLoS One 12, no. 11 (2017): e0188707, 10.1371/journal.pone.0188707. 29190785 PMC5708643

[12] J. Zhong , Y. Shibata , C. Wu , et al., “Functional Non‐Uniformity of Periodontal Ligaments Tunes Mechanobiological Stimuli Across Soft‐ and Hard‐Tissue Interfaces,” Acta Biomaterialia 170 (2023): 240–249, 10.1016/j.actbio.2023.08.047. 37634832

[13] S. Nakatsu , Y. Yoshinaga , A. Kuramoto , et al., “Occlusal Trauma Accelerates Attachment Loss at the Onset of Experimental Periodontitis in Rats,” Journal of Periodontal Research 49, no. 3 (2014): 314–322, 10.1111/jre.12109. 23808820

[14] Y. Song and J. Chung , “Aging Aggravates Periodontal Inflammatory Responses and Alveolar Bone Resorption by *Porphyromonas gingivalis* Infection,” Current Issues in Molecular Biology 45, no. 8 (2023): 6593–6604, 10.3390/cimb45080416. 37623235 PMC10453897

[15] T. Chen , J. Li , L. A. Cordova , et al., “A WNT Protein Therapeutic Improves the Bone‐Forming Capacity of Autografts From Aged Animals,” Scientific Reports 8, no. 1 (2018): 119, 10.1038/s41598-017-18375-x. 29311710 PMC5758817

[16] A. Mavropoulos , R. Rizzoli , and P. Ammann , “Different Responsiveness of Alveolar and Tibial Bone to Bone Loss Stimuli,” Journal of Bone and Mineral Research 22, no. 3 (2007): 403–410, 10.1359/jbmr.061208. 17181394

[17] H. Ahn , W. Park , S. H. Choi , N. Hong , J. Huh , and S. Jung , “Effect of Anti‐Sclerostin Antibody on Orthodontic Tooth Movement in Ovariectomized Rats,” Progress in Orthodontics 25, no. 1 (2024): 45, 10.1186/s40510-024-00544-0. 39581932 PMC11586325

[18] K. A. Carpenter , D. O. Alkhatib , B. A. Dulion , et al., “Sclerostin Antibody Improves Alveolar Bone Quality in the Hyp Mouse Model of X‐Linked Hypophosphatemia (XLH),” International Journal of Oral Science 15, no. 1 (2023): 47, 10.1038/s41368-023-00252-1. 37813865 PMC10562382

[19] C. C. Marcantonio , G. H. Perles , M. E. S. Lopes , et al., “Influence of Anti‐Sclerostin Monoclonal Antibody in the Repair of Post‐Extraction Sockets of Ovariectomized Rats,” Archives of Oral Biology 162 (2024): 105962, 10.1016/j.archoralbio.2024.105962. 38569446

[20] H. H. Sung , H. H. Kwon , C. Stephan , et al., “Sclerostin Antibody Enhances Implant Osseointegration in Bone With Col1a1 Mutation,” Bone 186 (2024): 117167, 10.1016/j.bone.2024.117167. 38876270 PMC11243590

[21] H. Turkkahraman , S. Flanagan , T. Zhu , et al., “Sclerostin Antibody Corrects Periodontal Disease in Type 2 Diabetic Mice,” JCI Insight 9, no. 16 (2024): 181940, 10.1172/jci.insight.181940. PMC11343605 39171525

[22] S. S. Huja and F. M. Beck , “Bone Remodeling in Maxilla, Mandible, and Femur of Young Dogs,” Anatomical Record 291, no. 1 (2008): 1–5, 10.1002/ar.20619. 18085627

[23] L. E. Randall , F. M. Beck , and S. S. Huja , “Bone Remodeling Surrounding Primary Teeth in Skeletally Immature Dogs,” Angle Orthodontist 81, no. 6 (2011): 931–937, 10.2319/021611-114.1. 21631293 PMC8903844

[24] S. O. Akintoye , T. Lam , S. Shi , J. Brahim , M. T. Collins , and P. G. Robey , “Skeletal Site‐Specific Characterization of Orofacial and Iliac Crest Human Bone Marrow Stromal Cells in Same Individuals,” Bone 38, no. 6 (2006): 758–768, 10.1016/j.bone.2005.10.027. 16403496

[25] S. Tsuchida and T. Nakayama , “Recent Clinical Treatment and Basic Research on the Alveolar Bone,” Biomedicine 11, no. 3 (2023): 843, 10.3390/biomedicines11030843. PMC10044990 36979821

[26] J. Y. Wang , L. Huo , R. Q. Yu , N. J. Rao , W. W. Lu , and L. W. Zheng , “Skeletal Site‐Specific Response of Jawbones and Long Bones to Surgical Interventions in Rats Treated With Zoledronic Acid,” BioMed Research International 2019 (2019): 5138175, 10.1155/2019/5138175. 31930124 PMC6942746

[27] M. Barczyk , A. I. Bolstad , and D. Gullberg , “Role of Integrins in the Periodontal Ligament: Organizers and Facilitators,” Periodontology 2000 63, no. 1 (2013): 29–47, 10.1111/prd.12027. 23931052 PMC3791550

[28] Y. Liang , Z. Hu , B. Chang , and X. Liu , “Quantitative Characterizations of the Sharpey's Fibers of Rat Molars,” Journal of Periodontal Research 55, no. 2 (2020): 307–314, 10.1111/jre.12716. 31788804 PMC7082191

[29] N. Odagaki , Y. Ishihara , Z. Wang , et al., “Role of Osteocyte‐PDL Crosstalk in Tooth Movement via SOST/Sclerostin,” Journal of Dental Research 97, no. 12 (2018): 1374–1382, 10.1177/0022034518771331. 29863952

[30] J. H. Lee , H. J. Kim , and J. H. Yun , “Three‐Dimensional Microstructure of Human Alveolar Trabecular Bone: A Micro‐Computed Tomography Study,” Journal of Periodontal & Implant Science 47, no. 1 (2017): 20, 10.5051/jpis.2017.47.1.20. 28261521 PMC5332332

[31] C. Son , M. S. Choi , and J. C. Park , “Different Responsiveness of Alveolar Bone and Long Bone to Epithelial‐Mesenchymal Interaction‐Related Factor,” JBMR Plus 4, no. 8 (2020): e10382, 10.1002/jbm4.10382. 32803111 PMC7422712

[32] H. Enomoto , S. Shiojiri , K. Hoshi , et al., “Induction of Osteoclast Differentiation by Runx2 Through Receptor Activator of Nuclear Factor‐κB Ligand (RANKL) and Osteoprotegerin Regulation and Partial Rescue of Osteoclastogenesis in Runx2^−/−^ Mice by RANKL Transgene,” Journal of Biological Chemistry 278, no. 26 (2003): 23971–23977, 10.1074/jbc.M302457200. 12697767

[33] Y. Ren , X. Han , S. P. Ho , et al., “Removal of SOST or Blocking Its Product Sclerostin Rescues Defects in the Periodontitis Mouse Model,” FASEB Journal 29, no. 7 (2015): 2702–2711, 10.1096/fj.14-265496. 25757567 PMC4478802

[34] S. Kuroda , R. Wazen , P. Moffatt , E. Tanaka , and A. Nanci , “Mechanical Stress Induces Bone Formation in the Maxillary Sinus in a Short‐Term Mouse Model,” Clinical Oral Investigations 17, no. 1 (2013): 131–137, 10.1007/s00784-012-0686-4. 22373776

[35] H. Seddiqi , J. Klein‐Nulend , and J. Jin , “Osteocyte Mechanotransduction in Orthodontic Tooth Movement,” Current Osteoporosis Reports 21, no. 6 (2023): 731–742, 10.1007/s11914-023-00826-2. 37792246 PMC10724326

[36] A. Phanrungsuwan , M. B. Chavez , L. A. Eltilib , et al., “Disparate Effects of Sclerostin Deletion on Alveolar Bone and Cellular Cementum in Mice,” Journal of Periodontology 96, no. 1 (2025): 82–96, 10.1002/JPER.24-0025. 39012429 PMC11735692

[37] G. S. Chatzopoulos , Z. Jiang , N. Marka , and L. F. Wolff , “Periodontal Disease, Tooth Loss, and Systemic Conditions: An Exploratory Study,” International Dental Journal 74, no. 2 (2024): 207–215, 10.1016/j.identj.2023.08.002. 37833208 PMC10988265

[38] T. S. Miranda , M. H. Napimoga , M. Feres , et al., “Antagonists of Wnt/Beta‐Catenin Signalling in the Periodontitis Associated With Type 2 Diabetes and Smoking,” Journal of Clinical Periodontology 45, no. 3 (2018): 293–302, 10.1111/jcpe.12854. 29243300

[39] Y. Li , L. A. Jacox , S. H. Little , and C. C. Ko , “Orthodontic Tooth Movement: The Biology and Clinical Implications,” Kaohsiung Journal of Medical Sciences 34, no. 4 (2018): 207–214, 10.1016/j.kjms.2018.01.007. 29655409 PMC11915602

[40] Y. Yu , J. Mu , Z. Fan , et al., “Insulin‐Like Growth Factor 1 Enhances the Proliferation and Osteogenic Differentiation of Human Periodontal Ligament Stem Cells via ERK and JNK MAPK Pathways,” Histochemistry and Cell Biology 137, no. 4 (2012): 513–525, 10.1007/s00418-011-0908-x. 22227802

[41] N. Ziegler , A. Alonso , T. Steinberg , et al., “Mechano‐Transduction in Periodontal Ligament Cells Identifies Activated States of MAP‐Kinases p42/44 and p38‐Stress Kinase as a Mechanism for MMP‐13 Expression,” BMC Cell Biology 11, no. 1 (2010): 10, 10.1186/1471-2121-11-10. 20109185 PMC2824740

[42] Y. Jiang , Y. Guan , Y. Lan , et al., “Mechanosensitive Piezo1 in Periodontal Ligament Cells Promotes Alveolar Bone Remodeling During Orthodontic Tooth Movement,” Frontiers in Physiology 12 (2021): 767136, 10.3389/fphys.2021.767136. 34880779 PMC8645976

[43] M. Rizk , C. Niederau , A. Florea , et al., “Periodontal Ligament and Alveolar Bone Remodeling During Long Orthodontic Tooth Movement Analyzed by a Novel User‐Independent 3D‐Methodology,” Scientific Reports 13, no. 1 (2023): 19919, 10.1038/s41598-023-47386-0. 37964111 PMC10646115

[44] R. Shu , D. Bai , T. Sheu , et al., “Sclerostin Promotes Bone Remodeling in the Process of Tooth Movement,” PLoS One 12, no. 1 (2017): e0167312, 10.1371/journal.pone.0167312. 28081119 PMC5230762

[45] X. Tu , Y. Rhee , K. W. Condon , et al., “Sost Downregulation and Local Wnt Signaling Are Required for the Osteogenic Response to Mechanical Loading,” Bone 50, no. 1 (2012): 209–217, 10.1016/j.bone.2011.10.025. 22075208 PMC3246572

[46] D. Cheng , X. Yuan , Z. Ma , et al., “Canonical Wnt Pathway Enhanced Dental Pulp Mesenchymal Stem Cells‐Mediated Cementum Regeneration,” Journal of Oral Rehabilitation 52, no. 10 (2025): 1571–1582, 10.1111/joor.14001. 40369784

[47] C. Nottmeier , N. Liao , A. Simon , et al., “Wnt1 Promotes Cementum and Alveolar Bone Growth in a Time‐Dependent Manner,” Journal of Dental Research 100, no. 13 (2021): 1501–1509, 10.1177/00220345211012386. 34009051 PMC8649456

[48] Y. Ono , M. Kaku , L. Thant , et al., “Wnt/Beta‐Catenin Promotes Cementum Apposition in Periodontal Regeneration,” Journal of Dental Research 104, no. 2 (2025): 183–192, 10.1177/00220345241286490. 39586793 PMC11752650

[49] Y. Ding , J. Luan , H. S. Malmstrom , X. Luan , and T. G. H. Diekwisch , “Mir‐27 Promotes Periodontal Regeneration via Osteogenesis/Angiogenesis,” Journal of Dental Research (2025): 220345251366279, 10.1177/00220345251366279. PMC13547813 41102998

[50] L. Ma , M. Li , G. Xuan , and Y. Dai , “METTL14‐Mediated m6A RNA Methylation Promotes the Osteogenic Differentiation of pPDLSCs by Regulating WNT3A,” Odontology 113, no. 4 (2025): 1701–1711, 10.1007/s10266-025-01097-2. 40249476

[51] D. Tan , Q. Li , Z. Chen , et al., “YTHDC1 Modulates the Osteogenic Capacity of hPDLSCs via Wnt/Beta‐Catenin Signalling Pathway for the Treatment of Bone Defects in Osteoporosis Rats,” Cell Proliferation 58, no. 8 (2025): e70020, 10.1111/cpr.70020. 40097906 PMC12336452

[52] P. E. Chang , S. Li , H. Y. Kim , D. J. Lee , Y. J. Choi , and H. S. Jung , “BBS7‐SHH Signaling Activity Regulates Primary Cilia for Periodontal Homeostasis,” Frontiers in Cell and Developmental Biology 9 (2021): 796274, 10.3389/fcell.2021.796274. 34957122 PMC8703258

[53] M. Hampl , P. Cela , H. L. Szabo‐Rogers , et al., “Role of Primary Cilia in Odontogenesis,” Journal of Dental Research 96, no. 9 (2017): 965–974, 10.1177/0022034517713688. 28605602 PMC5524235

[54] K. Wang , C. Xu , X. Xie , et al., “Axin2+ PDL Cells Directly Contribute to New Alveolar Bone Formation in Response to Orthodontic Tension Force,” Journal of Dental Research 101, no. 6 (2022): 695–703, 10.1177/00220345211062585. 35001706 PMC9124907

[55] L. Grgurevic , R. Novak , G. Salai , et al., “Identification of Bone Morphogenetic Protein 4 in the Saliva After the Placement of Fixed Orthodontic Appliance,” Progress in Orthodontics 22, no. 1 (2021): 19, 10.1186/s40510-021-00364-6. 34250561 PMC8273045

[56] M. Wang , J. Fan , A. Wang , et al., “Effect of Local Application of Bone Morphogenetic Protein‐2 on Experimental Tooth Movement and Biological Remodeling in Rats,” Frontiers in Physiology 14 (2023): 1111857, 10.3389/fphys.2023.1111857. 37143931 PMC10151543

[57] S. Narasimhan , S. Al Kawas , S. R. Shetty , H. S. Al‐Daghestani , and R. Samsudin , “Impact of Hypoxia on Alveolar Bone Dynamics and Remodeling,” Heliyon 10, no. 23 (2024): e40868, 10.1016/j.heliyon.2024.e40868. 39717576 PMC11664270

[58] K. Ploysongsang , Y. Kobayashi , Y. Lu , Y. Niki , J. Chavanavesh , and K. Moriyama , “The Effects of Systemic and Sustained Hypoxia on Orthodontic Tooth Movement in Rats,” Scientific Reports 15, no. 1 (2025): 21468, 10.1038/s41598-025-07949-9. 40594863 PMC12219902

[59] A. Morse , F. C. Ko , M. M. McDonald , et al., “Increased Anabolic Bone Response in Dkk1 KO Mice Following Tibial Compressive Loading,” Bone 131 (2020): 115054, 10.1016/j.bone.2019.115054. 31521827

[60] X. Guan , Y. He , Z. Wei , et al., “Crosstalk Between Wnt/Beta‐Catenin Signaling and NF‐kappaB Signaling Contributes to Apical Periodontitis,” International Immunopharmacology 98 (2021): 107843, 10.1016/j.intimp.2021.107843. 34153668

[61] Y. Hu , Z. Wang , C. Fan , et al., “Human Gingival Mesenchymal Stem Cell‐Derived Exosomes Cross‐Regulate the Wnt/Beta‐Catenin and NF‐kappaB Signalling Pathways in the Periodontal Inflammation Microenvironment,” Journal of Clinical Periodontology 50, no. 6 (2023): 796–806, 10.1111/jcpe.13798. 36843393

[62] C. Kornsuthisopon , A. Chansaenroj , J. Manokawinchoke , K. A. Tompkins , N. Pirarat , and T. Osathanon , “Non‐Canonical Wnt Signaling Participates in Jagged1‐Induced Osteo/Odontogenic Differentiation in Human Dental Pulp Stem Cells,” Scientific Reports 12, no. 1 (2022): 7583, 10.1038/s41598-022-11596-9. 35534526 PMC9085777

[63] C. H. Alves , K. L. Russi , N. C. Rocha , et al., “Host‐Microbiome Interactions Regarding Peri‐Implantitis and Dental Implant Loss,” Journal of Translational Medicine 20, no. 1 (2022): 425, 10.1186/s12967-022-03636-9. 36138430 PMC9502891

[64] K. Turajane , G. Ji , Y. Chinenov , et al., “RNA‐Seq Analysis of Peri‐Implant Tissue Shows Differences in Immune, Notch, Wnt, and Angiogenesis Pathways in Aged Versus Young Mice,” JBMR Plus 5, no. 11 (2021): e10535, 10.1002/jbm4.10535. 34761143 PMC8567488

[65] Y. Li , X. Li , D. Guo , et al., “Immune Dysregulation and Macrophage Polarization in Peri‐Implantitis,” Frontiers in Bioengineering and Biotechnology 12 (2024): 1291880, 10.3389/fbioe.2024.1291880. 38347915 PMC10859439

[66] C. Ulm , G. D. Strbac , A. Stavropoulos , A. Esfandeyari , T. Dobsak , and K. Bertl , “Improved Access to the Bone Marrow Space by Multiple Perforations of the Alveolar Bundle Bone After Tooth Extraction—A Case Report,” Clinical and Experimental Dental Research 8, no. 1 (2022): 3–8, 10.1002/cre2.474. 34296542 PMC8874110

[67] Y. Fan , P. Lyu , R. Bi , et al., “Creating an Atlas of the Bone Microenvironment During Oral Inflammatory‐Related Bone Disease Using Single‐Cell Profiling,” eLife 12 (2023): 82537, 10.7554/eLife.82537. PMC9925051 36722472

[68] A. Hosoya , N. Shalehin , H. Takebe , et al., “Stem Cell Properties of Gli1‐Positive Cells in the Periodontal Ligament,” Journal of Oral Biosciences 62, no. 4 (2020): 299–305, 10.1016/j.job.2020.08.002. 32882366

[69] D. Zhang , W. Lin , S. Jiang , et al., “Lepr‐Expressing PDLSCs Contribute to Periodontal Homeostasis and Respond to Mechanical Force by Piezo1,” Advanced Science 10, no. 29 (2023): e2303291, 10.1002/advs.202303291. 37553778 PMC10582421

[70] Y. Chen , Y. Weng , J. Huang , et al., “Leptin Receptor (+) Stromal Cells Respond to Periodontitis and Attenuate Alveolar Bone Repair via CCRL2‐Mediated Wnt Inhibition,” Journal of Bone and Mineral Research 39, no. 5 (2024): 611–626, 10.1093/jbmr/zjae036. 38477792

[71] H. Todd , G. L. Galea , L. B. Meakin , et al., “Wnt16 Is Associated With Age‐Related Bone Loss and Estrogen Withdrawal in Murine Bone,” PLoS One 10, no. 10 (2015): e0140260, 10.1371/journal.pone.0140260. 26451596 PMC4599960

[72] M. Piemontese , M. Almeida , A. G. Robling , et al., “Old Age Causes De Novo Intracortical Bone Remodeling and Porosity in Mice,” JCI Insight 2, no. 17 (2017): 93771, 10.1172/jci.insight.93771. 28878136 PMC5621920

[73] M. Becerikli , H. Jaurich , J. Schira , et al., “Age‐Dependent Alterations in Osteoblast and Osteoclast Activity in Human Cancellous Bone,” Journal of Cellular and Molecular Medicine 21, no. 11 (2017): 2773–2781, 10.1111/jcmm.13192. 28444839 PMC5661248

[74] J. L. Roberts , G. Liu , D. N. Paglia , et al., “Deletion of Wnt5a in Osteoclasts Results in Bone Loss Through Decreased Bone Formation,” Annals of the New York Academy of Sciences 1463, no. 1 (2020): 45–59, 10.1111/nyas.14293. 31919867

[75] R. L. Tiwari , P. Mishra , N. Martin , et al., “A Wnt5a‐Cdc42 Axis Controls Aging and Rejuvenation of Hair‐Follicle Stem Cells,” Aging 13, no. 4 (2021): 4778–4793, 10.18632/aging.202694. 33629967 PMC7950224

[76] M. Almeida , L. Han , M. Martin‐Millan , et al., “Skeletal Involution by Age‐Associated Oxidative Stress and Its Acceleration by Loss of Sex Steroids,” Journal of Biological Chemistry 282, no. 37 (2007): 27285–27297, 10.1074/jbc.M702810200. 17623659 PMC3119455

[77] S. Iyer , E. Ambrogini , S. M. Bartell , et al., “FOXOs Attenuate Bone Formation by Suppressing Wnt Signaling,” Journal of Clinical Investigation 123, no. 8 (2013): 3409–3419, 10.1172/JCI68049. 23867625 PMC3726166

[78] S. Iyer , L. Han , S. M. Bartell , et al., “Sirtuin1 (Sirt1) Promotes Cortical Bone Formation by Preventing Beta‐Catenin Sequestration by FoxO Transcription Factors in Osteoblast Progenitors,” Journal of Biological Chemistry 289, no. 35 (2014): 24069–24078, 10.1074/jbc.M114.561803. 25002589 PMC4148840

[79] Q. Huang and J. Wang , “CBP‐Mediated FOXO4 Acetylation Facilitates Postmenopausal Osteoporosis (PMO) Progression Through the Inhibition of the Wnt/Beta‐Catenin Signaling Pathway,” Histology and Histopathology 39, no. 8 (2024): 1017–1024, 10.14670/HH-18-680. 38037460

[80] S. Ucer , S. Iyer , H. N. Kim , et al., “The Effects of Aging and Sex Steroid Deficiency on the Murine Skeleton Are Independent and Mechanistically Distinct,” Journal of Bone and Mineral Research 32, no. 3 (2017): 560–574, 10.1002/jbmr.3014. 27714847 PMC5340621

[81] R. L. Jilka , M. Almeida , E. Ambrogini , et al., “Decreased Oxidative Stress and Greater Bone Anabolism in the Aged, When Compared to the Young, Murine Skeleton With Parathyroid Hormone Administration,” Aging Cell 9, no. 5 (2010): 851–867, 10.1111/j.1474-9726.2010.00616.x. 20698835 PMC2958819

[82] Y. Xiong , Y. Zhang , F. Zhou , et al., “FOXO1 Differentially Regulates Bone Formation in Young and Aged Mice,” Cellular Signalling 99 (2022): 110438, 10.1016/j.cellsig.2022.110438. 35981656

[83] R. J. Pignolo , S. F. Law , and A. Chandra , “Bone Aging, Cellular Senescence, and Osteoporosis,” JBMR Plus 5, no. 4 (2021): e10488, 10.1002/jbm4.10488. 33869998 PMC8046105

[84] R. J. Pignolo and A. Chandra , “Insights Into Age‐Related Osteoporosis From Senescence‐Based Preclinical Models and Human Accelerated Aging Paradigms,” Mechanisms of Ageing and Development 224 (2025): 112025, 10.1016/j.mad.2025.112025. 39805505 PMC11938943

[85] T. Teissier , V. Temkin , R. D. Pollak , and L. S. Cox , “Crosstalk Between Senescent Bone Cells and the Bone Tissue Microenvironment Influences Bone Fragility During Chronological Age and in Diabetes,” Frontiers in Physiology 13 (2022): 812157, 10.3389/fphys.2022.812157. 35388291 PMC8978545

[86] J. Lehmann , R. Narcisi , N. Franceschini , et al., “WNT/Beta‐Catenin Signalling Interrupts a Senescence‐Induction Cascade in Human Mesenchymal Stem Cells That Restricts Their Expansion,” Cellular and Molecular Life Sciences 79, no. 2 (2022): 82, 10.1007/s00018-021-04035-x. 35048158 PMC8770385

[87] S. Jo , S. Weon , B. Nam , et al., “WNT16 Elevation Induced Cell Senescence of Osteoblasts in Ankylosing Spondylitis,” Arthritis Research & Therapy 23, no. 1 (2021): 301, 10.1186/s13075-021-02670-0. 34879876 PMC8653593

[88] K. Weng , Y. He , X. Weng , and Y. Yuan , “Exercise Alleviates Osteoporosis by Regulating the Secretion of the Senescent Associated Secretory Phenotype,” Bone 196 (2025): 117485, 10.1016/j.bone.2025.117485. 40216288

[89] Q. Geng , S. Wang , K. Heng , et al., “Astaxanthin Attenuates Irradiation‐Induced Osteoporosis in Mice by Inhibiting Oxidative Stress, Osteocyte Senescence, and SASP,” Food & Function 13, no. 22 (2022): 11770–11779, 10.1039/d2fo01673g. 36285709

[90] Q. Geng , H. Gao , R. Yang , K. Guo , and D. Miao , “Pyrroloquinoline Quinone Prevents Estrogen Deficiency‐Induced Osteoporosis by Inhibiting Oxidative Stress and Osteocyte Senescence,” International Journal of Biological Sciences 15, no. 1 (2019): 58–68, 10.7150/ijbs.25783. 30662347 PMC6329928

[91] B. Yu , J. Chang , Y. Liu , et al., “Wnt4 Signaling Prevents Skeletal Aging and Inflammation by Inhibiting Nuclear Factor‐kappaB,” Nature Medicine 20, no. 9 (2014): 1009–1017, 10.1038/nm.3586. PMC4159424 25108526

[92] W. Xu , Q. Lu , M. Qu , et al., “Wnt4 Regulates Bone Metabolism Through IKK‐NF‐kappaB and ROCK Signaling Under Occlusal Traumatic Periodontitis,” Journal of Periodontal Research 57, no. 3 (2022): 461–469, 10.1111/jre.12975. 35137408

[93] A. James , J. A. Hendrixson , I. Kadhim , et al., “Elevation of Master Autophagy Regulator Tfeb in Osteoblast Lineage Cells Increases Bone Mass and Strength,” JCI Insight 10, no. 17 (2025): 191688, 10.1172/jci.insight.191688. PMC12487674 40728889

[94] M. Onal , “TFEB‐Mediated Autophagy Stimulation as an Anabolic Strategy for Bone: Insights From TFEB Activation in the Osteoblast Lineage,” Autophagy Reports 4, no. 1 (2025): 2596422, 10.1080/27694127.2025.2596422. 41377164 PMC12688215

[95] M. Onal , M. Piemontese , J. Xiong , et al., “Suppression of Autophagy in Osteocytes Mimics Skeletal Aging,” Journal of Biological Chemistry 288, no. 24 (2013): 17432–17440, 10.1074/jbc.M112.444190. 23645674 PMC3682543

[96] M. Piemontese , M. Onal , J. Xiong , et al., “Low Bone Mass and Changes in the Osteocyte Network in Mice Lacking Autophagy in the Osteoblast Lineage,” Scientific Reports 6 (2016): 24262, 10.1038/srep24262. 27064143 PMC4827128

[97] A. Resende‐Coelho , M. M. Ali , A. James , et al., “Mitochondrial Oxidative Stress or Decreased Autophagy in Osteoblast Lineage Cells Is Not Sufficient to Mimic the Deleterious Effects of Aging on Bone Mechanoresponsiveness,” Aging 17, no. 3 (2025): 610–629, 10.18632/aging.206213. 40105873 PMC11984430

[98] L. Chen , Y. Yang , J. Bao , et al., “Autophagy Negative‐Regulating Wnt Signaling Enhanced Inflammatory Osteoclastogenesis From Pre‐OCs In Vitro,” Biomedicine & Pharmacotherapy 126 (2020): 110093, 10.1016/j.biopha.2020.110093. 32199225

[99] H. Kato , X. Han , H. Yamaza , et al., “Direct Effects of Mitochondrial Dysfunction on Poor Bone Health in Leigh Syndrome,” Biochemical and Biophysical Research Communications 493, no. 1 (2017): 207–212, 10.1016/j.bbrc.2017.09.045. 28899781

[100] H. Zhang , R. Zhao , X. Wang , et al., “Interruption of Mitochondrial Symbiosis Is Associated With the Development of Osteoporosis,” Frontiers in Endocrinology 16 (2025): 1488489, 10.3389/fendo.2025.1488489. 39963284 PMC11830588

[101] X. Sun , Y. Mao , P. Dai , et al., “Mitochondrial Dysfunction Is Involved in the Aggravation of Periodontitis by Diabetes,” Journal of Clinical Periodontology 44, no. 5 (2017): 463–471, 10.1111/jcpe.12711. 28207937

[102] T. Xu , Q. Dong , Y. Luo , et al., “Correction to: *Porphyromonas gingivalis* Infection Promotes Mitochondrial Dysfunction Through Drp1‐Dependent Mitochondrial Fission in Endothelial Cells,” International Journal of Oral Science 14, no. 1 (2022): 3, 10.1038/s41368-021-00153-1. 35022392 PMC8755809

[103] Y. Du , J. Zheng , B. Xu , C. Peng , and K. Yang , “Piezo1 Participates in the Tension‐Driven Osteogenic Differentiation of Periodontal Ligament Stem Cells,” BMC Oral Health 25, no. 1 (2025): 1155, 10.1186/s12903-025-06427-y. 40653466 PMC12257747

[104] T. Hasegawa , T. Ouchi , R. Kurashima , et al., “Piezo1 Negatively Regulates Proliferation, but Enhances Mineralization in Human Cementoblasts,” Journal of Oral Biosciences 67, no. 4 (2025): 100705, 10.1016/j.job.2025.100705. 41274689

[105] X. Wang , B. Xie , Y. Guo , et al., “Agonism of PIEZO1 Prevents Aggravated Periodontitis With Traumatic Occlusion via MAPK Signaling Pathway,” iScience 28, no. 11 (2025): 113688, 10.1016/j.isci.2025.113688. 41234768 PMC12605257

[106] S. Shindo , S. Nakamura , A. Hawthorne , et al., “Piezo1 Protects Against Inflammatory Bone Loss via a Unique Ca(2+)‐Independent Mechanism in Osteoclasts,” Frontiers in Immunology 16 (2025): 1661538, 10.3389/fimmu.2025.1661538. 41080556 PMC12507952

[107] Y. Gao , E. Huang , H. Zhang , et al., “Crosstalk Between Wnt/Beta‐Catenin and Estrogen Receptor Signaling Synergistically Promotes Osteogenic Differentiation of Mesenchymal Progenitor Cells,” PLoS One 8, no. 12 (2013): e82436, 10.1371/journal.pone.0082436. 24340027 PMC3855436

[108] A. Liedert , C. Nemitz , M. Haffner‐Luntzer , F. Schick , F. Jakob , and A. Ignatius , “Effects of Estrogen Receptor and Wnt Signaling Activation on Mechanically Induced Bone Formation in a Mouse Model of Postmenopausal Bone Loss,” International Journal of Molecular Sciences 21, no. 21 (2020): 8301, 10.3390/ijms21218301. 33167497 PMC7663944

[109] S. Moverare‐Skrtic , J. Wu , P. Henning , et al., “The Bone‐Sparing Effects of Estrogen and WNT16 Are Independent of Each Other,” Proceedings of the National Academy of Sciences of the United States of America 112, no. 48 (2015): 14972–14977, 10.1073/pnas.1520408112. 26627248 PMC4672787

[110] D. Zhang , M. Hu , T. Chu , et al., “Sclerostin Antibody Prevented Progressive Bone Loss in Combined Ovariectomized and Concurrent Functional Disuse,” Bone 87 (2016): 161–168, 10.1016/j.bone.2016.02.005. 26868528 PMC4862887

[111] C. MacNabb , D. Patton , and J. S. Hayes , “Sclerostin Antibody Therapy for the Treatment of Osteoporosis: Clinical Prospects and Challenges,” Journal of Osteoporosis 2016 (2016): 6217286, 10.1155/2016/6217286. 27313945 PMC4899597

[112] F. Cosman , D. B. Crittenden , J. D. Adachi , et al., “Romosozumab Treatment in Postmenopausal Women With Osteoporosis,” New England Journal of Medicine 375, no. 16 (2016): 1532–1543, 10.1056/NEJMoa1607948. 27641143

[113] N. R. Momesso , A. C. Z. Bacelar‐Marcolino , C. C. Biguetti , R. C. Ortiz , E. Ervolino , and M. A. Matsumoto , “Comparative Effects of Ovariectomy and Chemically Induced Menopause on Alveolar Bone Healing in Zoledronate‐Treated Female Mice,” Bone 201 (2025): 117655, 10.1016/j.bone.2025.117655. 41005449

[114] C. C. Furlan , A. R. Freire , B. C. Ferreira‐Pileggi , et al., “Does Ovariectomy Affect the Mechanics of the Mandibular Alveolar Bone Structure of Wistar Rats Subjected to Tooth Loss and Modified Diet?—A FEA Study,” Biology‐Basel 13, no. 11 (2024): 906, 10.3390/biology13110906. 39596861 PMC11592268

[115] H. Chen , X. Xu , M. Liu , et al., “Sclerostin Antibody Treatment Causes Greater Alveolar Crest Height and Bone Mass in an Ovariectomized Rat Model of Localized Periodontitis,” Bone 76 (2015): 141–148, 10.1016/j.bone.2015.04.002. 25868799

[116] B. Gharibi , M. S. Ghuman , G. Cama , S. C. F. Rawlinson , A. E. Grigoriadis , and F. J. Hughes , “Site‐Specific Differences in Osteoblast Phenotype, Mechanical Loading Response and Estrogen Receptor‐Related Gene Expression,” Molecular and Cellular Endocrinology 477 (2018): 140–147, 10.1016/j.mce.2018.06.011. 29928929

[117] Y. Yao , F. Kauffmann , S. Maekawa , et al., “Sclerostin Antibody Stimulates Periodontal Regeneration in Large Alveolar Bone Defects,” Scientific Reports 10, no. 1 (2020): 16217, 10.1038/s41598-020-73026-y. 33004873 PMC7530715

[118] S. H. Yu , J. Hao , T. Fretwurst , et al., “Sclerostin‐Neutralizing Antibody Enhances Bone Regeneration Around Oral Implants,” Tissue Engineering, Part A 24 (2018): 1672–1679, 10.1089/ten.TEA.2018.0013. 29921173 PMC6916116

[119] E. Ergunol , R. Semsi , D. Dayanir , et al., “Sclerostin Antibody Promotes Alveolar Bone Regeneration After Tooth Extraction,” Biomolecules & Biomedicine 2025 (2025): 12999, 10.17305/bb.2025.12999. PMC13130743 41123196

[120] K. G. Saag , J. Petersen , M. L. Brandi , et al., “Romosozumab or Alendronate for Fracture Prevention in Women With Osteoporosis,” New England Journal of Medicine 377, no. 15 (2017): 1417–1427, 10.1056/NEJMoa1708322. 28892457

[121] S. Gonzalez‐Salvatierra , C. Garcia‐Fontana , J. Lacal , et al., “Cardioprotective Function of Sclerostin by Reducing Calcium Deposition, Proliferation, and Apoptosis in Human Vascular Smooth Muscle Cells,” Cardiovascular Diabetology 22, no. 1 (2023): 301, 10.1186/s12933-023-02043-8. 37919715 PMC10623848

[122] J. Golledge and S. Thanigaimani , “Role of Sclerostin in Cardiovascular Disease,” Arteriosclerosis, Thrombosis, and Vascular Biology 42, no. 7 (2022): e187–e202, 10.1161/ATVBAHA.122.317635. 35546488

[123] M. Liu , P. Kurimoto , J. Zhang , et al., “Sclerostin and DKK1 Inhibition Preserves and Augments Alveolar Bone Volume and Architecture in Rats With Alveolar Bone Loss,” Journal of Dental Research 97, no. 9 (2018): 1031–1038, 10.1177/0022034518766874. 29617179

[124] M. Florio , K. Gunasekaran , M. Stolina , et al., “A Bispecific Antibody Targeting Sclerostin and DKK‐1 Promotes Bone Mass Accrual and Fracture Repair,” Nature Communications 7 (2016): 11505, 10.1038/ncomms11505. PMC4894982 27230681

[125] P. C. Witcher , S. E. Miner , D. J. Horan , et al., “Sclerostin Neutralization Unleashes the Osteoanabolic Effects of Dkk1 Inhibition,” JCI Insight 3, no. 11 (2018): 98673, 10.1172/jci.insight.98673. 29875318 PMC6124404

[126] J. Y. Ko , F. S. Wang , W. S. Lian , et al., “Dickkopf‐1 (DKK1) Blockade Mitigates Osteogenesis Imperfecta (OI) Related Bone Disease,” Molecular Medicine 30, no. 1 (2024): 66, 10.1186/s10020-024-00838-3. 38773377 PMC11106911

[127] N. Tuysuz , L. van Bloois , S. Brink , et al., “Lipid‐Mediated Wnt Protein Stabilization Enables Serum‐Free Culture of Human Organ Stem Cells,” Nature Communications 8 (2017): 14578, 10.1038/ncomms14578. PMC5343445 28262686

[128] G. Rawadi , B. Vayssiere , F. Dunn , R. Baron , and S. Roman‐Roman , “BMP‐2 Controls Alkaline Phosphatase Expression and Osteoblast Mineralization by a Wnt Autocrine Loop,” Journal of Bone and Mineral Research 18, no. 10 (2003): 1842–1853, 10.1359/jbmr.2003.18.10.1842. 14584895

[129] L. Wei , F. Teng , L. Deng , et al., “Periodontal Regeneration Using Bone Morphogenetic Protein 2 Incorporated Biomimetic Calcium Phosphate in Conjunction With Barrier Membrane: A Pre‐Clinical Study in Dogs,” Journal of Clinical Periodontology 46, no. 12 (2019): 1254–1263, 10.1111/jcpe.13195. 31518453 PMC6899729

[130] S. Jiang , T. Liu , G. Wu , et al., “BMP2‐Functionalized Biomimetic Calcium Phosphate Graft Promotes Alveolar Defect Healing During Orthodontic Tooth Movement in Beagle Dogs,” Frontiers in Bioengineering and Biotechnology 8 (2020): 517, 10.3389/fbioe.2020.00517. 32548104 PMC7272671

[131] J. W. Nam , S. Khureltogtokh , H. M. Choi , A. R. Lee , Y. B. Park , and H. J. Kim , “Randomised Controlled Clinical Trial of Augmentation of the Alveolar Ridge Using Recombinant Human Bone Morphogenetic Protein 2 With Hydroxyapatite and Bovine‐Derived Xenografts: Comparison of Changes in Volume,” British Journal of Oral and Maxillofacial Surgery 55, no. 8 (2017): 822–829, 10.1016/j.bjoms.2017.07.017. 28864147

[132] R. J. Miron , M. Dard , and M. Weinreb , “Enamel Matrix Derivative, Inflammation and Soft Tissue Wound Healing,” Journal of Periodontal Research 50, no. 5 (2015): 555–569, 10.1111/jre.12245. 25418917

[133] M. Kitamura , M. Yamashita , K. Miki , et al., “An Exploratory Clinical Trial to Evaluate the Safety and Efficacy of Combination Therapy of REGROTH(R) and Cytrans(R) Granules for Severe Periodontitis With Intrabony Defects,” Regenerative Therapy 21 (2022): 104–113, 10.1016/j.reth.2022.06.001. 35785043 PMC9234541

[134] A. Sculean , P. Windisch , G. C. Chiantella , N. Donos , M. Brecx , and E. Reich , “Treatment of Intrabony Defects With Enamel Matrix Proteins and Guided Tissue Regeneration. A Prospective Controlled Clinical Study,” Journal of Clinical Periodontology 28, no. 5 (2001): 397–403, 10.1034/j.1600-051x.2001.028005397.x. 11350501

[135] S. P. De Ry , A. Roccuzzo , N. P. Lang , A. Sculean , and G. E. Salvi , “Long‐Term Clinical Outcomes of Periodontal Regeneration With Enamel Matrix Derivative: A Retrospective Cohort Study With a Mean Follow‐Up of 10 Years,” Journal of Periodontology 93, no. 4 (2022): 548–559, 10.1002/JPER.21-0347. 34258767 PMC9373923

